# Research on different modes of energy conservation and emission reduction: A differential game model based on carbon trading perspective

**DOI:** 10.1371/journal.pone.0309968

**Published:** 2024-09-04

**Authors:** Xueli Zhu

**Affiliations:** Business School, Shandong Management University, Jinan, China; Guangdong University of Finance and Economics, CHINA

## Abstract

In recent years, due to global climate change, increasing resource scarcity, and environmental constraints, countries have prioritized energy conservation and emissions reduction. However, enterprises are primarily responsible for energy saving and emissions reduction. To encourage industrial enterprises to engage in energy conservation and emissions reduction, high-carbon enterprises must purchase carbon emission rights from low-carbon counterparts. Common modes of energy conservation and emission reduction of industrial enterprises include reducing production scale, improving energy utilization efficiency, and expanding renewable energy. This article constructs three differential game models to identify the applicable scope of various energy conservation and emission reduction strategies, comparing and analyzing the equilibrium results. The study concludes that when the cost of changing the production mode and the income obtained from the production of unit product is large, the low-carbon enterprise can obtain the maximum benefit by reducing the production scale mode. Otherwise, low carbon enterprises can be maximized through improving energy efficiency mode. For both low-carbon and high-carbon enterprises, reducing production scale is the fastest way to enhance efficiency when the costs of energy conservation and emission reduction are substantial.

## 1. Introduction

Enterprises are seen as key players in mitigating the issues of extreme weather events, rising sea levels, melting glaciers, and loss of biodiversity due to global warming, as they are one of the major sources of global greenhouse gas emissions. Carbon emissions are a significant contributor to climate warming, which is a primary cause of heat waves, with enterprise production and operation being the main sources of carbon emissions.

Since the United Nations Framework Convention on Climate Change (UNFCCC) in 1992, various international agreements and protocols (e.g., Kyoto Protocol, Paris Agreement) have aimed to limit and reduce greenhouse gas emissions. These agreements often set targets for individual countries, which are passed on to domestic industries and businesses. Many countries and regions have implemented mandatory environmental protection regulations that require companies to reduce pollutant emissions and improve energy efficiency.

Enterprises can be classified as either high or low-carbon depending on the amount of carbon emissions produced during their activities. High-carbon enterprises typically rely on energy-intensive production methods and face greater risks and responsibilities regarding carbon emissions. Conversely, low-carbon enterprises are committed to reducing carbon emissions in their production and operational activities.

Carbon trading is a flexible mechanism designed to reduce overall greenhouse gas emissions and incentivize enterprises to decrease their carbon footprint. It encourages high-carbon enterprises to actively explore and adopt low-carbon technologies and innovations, while also providing economic benefits for low-carbon businesses to maintain low emissions [1]. If high-carbon enterprises exceed their allocated carbon emission limits, they must purchase additional emission rights, increasing their operating costs, which acts as an economic incentive to reduce emissions.

As awareness of environmental protection increases, consumers begin to favor companies that implement environmentally friendly policies. Having a well-structured energy efficiency strategy can help a company establish a competitive advantage in the marketplace and attract investors, customers, and partners. Therefore, taking energy efficiency and emission reduction measures now can help businesses better prepare for the energy and environmental challenges they may face in the future, with resources becoming increasingly scarce and environmental constraints increasing [2]. The significance of carbon trading lies in its ability to provide a market mechanism that incentivizes enterprises to promote the development of the economy toward a low-carbon direction.

Drawing upon the research lacunae highlighted in prior studies, my research contribution and innovation can be succinctly summarized along several lines. Firstly, my study addresses this clear void with a comprehensive synthesis that integrates the dimensions of optimizing production scales, enhancing energy efficiency, and expanding renewable energy within the purview of carbon trading. This investigation breaks new ground by analyzing the dynamic interplay between these elements, aimed at significantly augmenting the efficacy of carbon trading as a strategy for energy conservation and emissions reduction.

Secondly, regarding the role of technological innovation, this study transcends the existing boundaries of study. While not concentrating on specific technologies, it meticulously examines the scalability and integration of state-of-the-art technologies within the energy system and market, especially from the perspective of carbon trading. Employing analytical methods such as game theory and optimization, this study conducts a comprehensive analysis on the large-scale implementation of innovative technologies, thereby bolstering their contributions to the carbon trading market and cultivating a more sustainable energy infrastructure.

Lastly, in the realm of operations management within carbon trading, this study ventures into a largely unexplored territory by elucidating how operational efficiency and management behaviors can be improved to meet carbon trading objectives. This inquiry thoroughly explores how entities participating in the carbon market can refine their operations, leverage technological innovations, and adhere to constructive policies, thereby enhancing their carbon trading efficiency and contributing to emission reductions.

This article suggests integrating carbon trading with energy conservation and emissions reduction in industrial enterprises. This strategy mandates that high-carbon enterprises buy carbon emission rights from their low-carbon counterparts. In order to derive the appropriate energy saving and emission reduction paths for low-carbon and high-carbon enterprises to choose under carbon trading, this article constructs a game mode with three modes: reducing production scale, improving energy efficiency or expanding renewable energy based on carbon trading. The equilibrium result is then derived using the Hamilton-Jacobi-Bellman (HJB) equation. Finally the article compares the equilibrium results under different modes. This comparison offers guidance for both low-carbon and high-carbon enterprises in selecting suitable strategies for energy conservation and emissions reduction.

In the process of energy conservation and emission reduction in industrial enterprises, there are three ways of energy conservation and emission reduction.

1. Reducing production scale. Reducing production scale is a common method for industrial enterprises to conserve energy and reduce emissions. This approach helps reduce energy and resource consumption, leading to lower carbon emissions. A smaller production scale requires fewer resources, thereby reducing the demand for fuel and electricity and subsequently lowering carbon emissions.
2. Improving energy efficiency. Improving energy utilization efficiency is a popular strategy among industrial enterprises for energy conservation and emission reduction. This enables more effective energy use in production, reduced energy waste, and lower carbon emissions. Various measures, including technical improvements, can achieve this. Establishing an effective energy management system, conducting energy monitoring and data analysis, and targeting energy-intensive areas for improvement are recommended to optimize energy consumption [3].
3. Expanding renewable energy. Using renewable energy is a widespread strategy for industrial enterprises to conserve energy and cut emissions. Renewable sources like solar, wind, water, and bioenergy offer clean and sustainable alternatives to conventional fossil fuels. Increasing renewable energy usage reduces reliance on conventional energy sources, lowers costs, and significantly cuts carbon emissions, benefiting environmental protection and climate change [4]. However, enterprises must weigh various factors, such as technology maturity, economic feasibility, and energy supply stability, when increasing renewable energy usage. Careful planning and execution are essential for the effective use of renewable energy.

This paper advances the differential game model for energy conservation and emissions reduction research, moving beyond merely adopting the modes of other scholars. The mode incorporates carbon trading into the differential game and clearly depicts the interactions between low-carbon and high-carbon enterprises. This enables analysis of market mechanisms, quantification of strategic behaviors, and development of carbon emissions reduction dynamics.

This study holds significant practical importance. Firstly, as major contributors to greenhouse gas emissions, businesses are at the forefront of mitigating climate change through various means, including reducing production scale, enhancing energy efficiency, and expanding the use of renewable energy. The urgent need to curb the negative impacts of climate change such as extreme weather events, rising sea levels, melting glaciers, and biodiversity loss is pressing. Against this backdrop, carbon trading becomes a crucial mechanism by intertwining economic incentives with environmental goals, driving both high and low-carbon enterprises towards more sustainable operational models. Moreover, it encourages the adoption of cleaner, more efficient technologies, directly addressing the core issue of whether businesses should reduce scale, enhance energy efficiency, or increase the usage of renewable energy. This study offers businesses a cost-effective way to comply with regulations. Within the carbon trading framework proposed by this research, making choices between reducing production scale, optimizing energy efficiency, or utilizing renewable energy turns into a strategic decision influenced by policy compliance and market dynamics. Lastly, the ever-changing scarcity of resources and environmental regulations demand that businesses adopt a proactive strategy. This research provides an economic incentive mechanism for current emission reductions, potentially alleviating the risks and costs associated with future resource constraints and stricter environmental policies. It involves finding a balance between expanding production scale, improving energy efficiency, and expanding the use of renewable energy. This not only relates to immediate interests but also concerns ensuring long-term viability and resilience in a low-carbon economy.

## 2. Literature review and hypothesis

### 2.1 Literature review

Energy conservation and emission reduction not only benefit the sustainable development of the economy but also significantly improve air quality and environmental protection. Some scholars have studied the effects of energy conservation and emissions reduction. This has been included to analyze the economic and environmental impacts of these practices. For instance, Wang et al. [5] explored how energy conservation and emissions reduction affect economic agglomeration; Xie et al. [6] examined the combined effects of energy conservation, decarbonization, and cost reduction on air pollutants. These studies primarily focused on how energy conservation and emissions reduction influence the economy and air quality.

Achieving energy conservation and emissions reduction requires efforts across multiple fronts, including operational management, technological innovation, and the enhancement of industrial policies. Each aspect will contribute uniquely to the process of energy conservation and emissions reduction.

In operation and management, ensuring effective energy use through reasonable planning and management measures is vital. This involves enhancing energy efficiency, minimizing waste, and optimizing supply and demand alignment. Several scholars have explored this topic, for instance, Xin et al. [7] analyzed effective pathways for the Henan steel industry to achieve energy savings and emissions reductions based on a model of steel demand and scrap steel recycling; Ma et al. [8] performed a macro-optimization of zero-energy consumption design solutions for residential buildings based on architectural prototypes; Zhang and Zhang [9] developed an optimization model for climate responsibility through cooperative game between the Chinese steel industry and consumers; Yu et al. [10] examined the pathways for reducing atmospheric pollutants in the Chinese electric power industry; Zhao et al. [11] optimized the distribution paths of electric vehicles within the urban fresh food cold chain; Wang and Wen [12] studied vehicle optimization in cold chain logistics to achieve lower carbon emissions; Lin et al. [13] optimized logistics sites through multi-objective optimization. These studies enhance energy conservation and emissions reduction efforts by optimizing energy management methods, system design, and improving monitoring and evaluation capabilities.

Technological innovation is vital for energy conservation and emission reduction. It necessitates greater investment in research and development for energy conservation and environmental protection, alongside breakthroughs in new, clean, and efficient energy technologies. This has been studied by several scholars. For instance, Chaurasiya et al. [14] explored energy saving and emission reduction via wind power technology. Liu and Lin [15] investigated the effects of China’s rail electrification on conserving energy and reducing emissions. Ma and Lin [16] analyzed the active utilization of digital technology for resource conservation and pollution reduction. Yang and Su [17] explored the positive impact of heat storage and energy dispatching characteristics of accumulators on the dynamic energy demand of marine generators. Zhang et al. [18] examined the future trends of terminal energy conservation in steel plants from a technical perspective. Lan et al. [19] examined how recovering waste heat from graphitization furnaces affects the potential to reduce pollutant emissions. Saboori and Jadid [20] analyzed integrated mobile battery charging as a strategy to reduce queues and costs for electric vehicle charging. Sheik et al. [21] explored controlling photovoltaic temperature with phase change materials (PCM) and nanoparticle phase change materials (NPCM) to enhance energy conversion efficiency. Agrawal et al. [22] improved energy use efficiency by testing discrete roughened panel solar air heaters’ thermofluid performance. These studies primarily concentrate on developing and applying new technologies to conserve energy and reduce emissions.

Formulating and adjusting industrial policies is crucial for energy conservation and emission reduction. The government can promote investment in energy-saving technologies by enterprises and individuals through incentive policies, tax breaks, preferential treatment, and financial support. Scholars have analyzed ways to achieve energy conservation and emission reduction through industrial economic policies. For example, Zhou et al. [23] examined the role of developing manufacturing value chains in advancing China’s energy conservation and emission reduction efforts. Yang et al. [24] analyzed whether Taiwan’s "2025 Nuclear-Free Homeland" policy could achieve its intended carbon emission reduction targets. Dinga and Wen [25] examined the impact of China’s green agreements on whether the Chinese cement industry can achieve carbon-neutral emissions by 2060. Chen and Ma [26] explored the impact of green investment on energy enterprises’ performance. Yuan et al. [27] examined the role of fiscal subsidies and tax incentives in guiding the development of electric vehicles. These studies address how value chains, investment, subsidies, and taxes affect energy conservation and emissions reduction.

In summary, operational management, technological innovation, and industrial policy play crucial roles in energy conservation and emission reduction. Their success depends on the collaboration of the government, businesses, and all societal sectors to foster sustainable energy development and protect the environment.

Some scholars have studied carbon trading. For example, Wang et al. [28] analyzed the location optimization of cold chain distribution centers for fresh agricultural products under carbon emission constraints. Gu et al. [29] investigated how carbon finance markets support environmental policies in promoting energy conservation and emissions reduction regionally. Jung et al. [30] explored the connection between carbon emission regulations, green boards, and corporate environmental responsibility. Sun and Gao [31] examined the ideal balance between carbon emission regulation and market structure. Liu et al. [32] utilized carbon emission rights to analyze unified strategies in carbon markets. Yadav et al. [33] argued that selecting projects with cross-demand price elasticity could reduce waste and carbon emissions, contributing to a sustainable supply chain. Jin et al. [34] studied how to optimize carbon emission reduction strategies during low-carbon power dispatch. Zhang et al. [35] investigated the complex formation and spatial correlations of carbon emission efficiency networks in the Yangtze River Economic Belt. Zhou and Zhao [36] examined the effects of renewable energy portfolio standards on carbon emission trading within China’s electricity market. These studies primarily focused on how carbon trading affects location optimization, corporate responsibility, market structure, and supply chains.

The existing literature encompasses a rich tapestry of research focusing on operational management, technological innovation, industrial policies, and carbon trading mechanisms as pathways to achieve energy conservation and emission reduction. However, there remain notable gaps in the collective understanding of how these disparate elements interconnect to form a cohesive energy conservation and carbon reduction strategy, particularly in light of scaling down production scales, enhancing energy efficiency, and expanding renewable energy sources in relation to carbon trading.

Firstly, while numerous studies have delineated optimization models, technological advancements, and policy interventions for energy conservation and emissions reduction, there appears to be a lack of comprehensive synthesis on how these dimensions can be collaboratively harnessed to enhance the efficacy of carbon trading as a pivotal energy conservation and emission reduction strategy. Specifically, the literature is scant on exploring the dynamic interplay between production scale adjustments, energy efficiency improvements, and the augmentation of renewable energy sources within the carbon trading framework to optimize the energy conservation and emission reduction outcomes.

Secondly, despite the recognition of technological innovation as a crucial driver for energy conservation and emission reduction, the literature has not sufficiently addressed the scalability and integration of these technologies within existing energy systems and markets, particularly from the perspective of carbon trading. This includes an in-depth analysis of how novel and emerging technologies can be feasibly implemented on a large scale to significantly contribute to the carbon trading market, thereby fostering a more sustainable energy landscape.

Thirdly, on the front of industrial policies, while there is substantial analysis of the potential impacts of policies on energy conservation and emissions reduction, there is a gap in understanding how these policies can be designed or adjusted to maximize the benefits of carbon trading. This involves a nuanced investigation into the policy frameworks that can effectively incentivize both the supply and demand sides of the carbon trading market, thereby driving a holistic approach to energy conservation and emission reduction across various sectors.

Lastly, the aspect of operational management, particularly in relation to carbon trading, has been under-explored in terms of how operational efficiencies and management practices can be optimized to support the objectives of carbon trading. This includes a detailed exploration of how entities participating in the carbon trading market can streamline their operations, leverage technological innovations, and adhere to supportive policies to enhance their carbon trading efficiency and contributions to emission reductions.

### 2.2 Hypothesis

This paper makes the following assumptions:

(1) High carbon enterprises need to purchase certain carbon emission rights from low carbon enterprises

In the carbon market, high carbon enterprises have the option to purchase carbon emission rights from low-carbon enterprises. This trading method is commonly used to regulate total emissions. In the carbon market, high carbon enterprises have the option to purchase carbon emission rights from low-carbon enterprises. Low-carbon enterprises can obtain additional carbon emission rights by implementing energy conservation and emission reduction measures, resulting in actual emissions that are lower than the allocated emission quota. However, high carbon companies may need to purchase additional carbon emission rights to compensate for exceeding their allocated emission quota. Additionally, low-carbon enterprises can benefit economically through carbon emission trading, which incentivizes them to continue achieving results in energy conservation and emission reduction, further promoting the development of low-carbon industries. The implementation of carbon emission trading must comply with relevant policies, regulations, and market mechanisms. The development of the carbon market requires government support and supervision to ensure fair, transparent, and effective transactions. Additionally, the carbon market must establish reliable monitoring, reporting, and verification mechanisms to ensure the authenticity and reliability of carbon emission rights.

(2) Improving energy efficiency can increase costs and benefits

Improving energy efficiency can have both costs and benefits, depending on the specific situation and implementation measures. Firstly, there may be increased costs due to technology investment. Implementing energy efficiency improvements requires investment in new equipment, technologies, or systems, which may lead to higher initial costs [37]. Secondly, there may be additional costs associated with training and management. Improving energy efficiency may require training employees and establishing effective energy management systems. These costs may increase. Additionally, many countries and regions have policies and incentive mechanisms supporting energy conservation and emission reduction, which may provide additional subsidies or benefits for improving energy efficiency. Fourth, improving energy efficiency can reduce carbon emissions of enterprises and enhance their environmental image and brand recognition. This may attract more environmentally conscious consumers and partners, further increasing benefits. Improving energy efficiency may involve initial costs and investments, but in the long run, it can bring cost savings, incentive benefits, brand value, and long-term competitiveness, which are beneficial for enterprises. Enterprises should comprehensively consider various factors and develop appropriate strategies to balance costs and benefits.

(3) Expanding the use of renewable energy sources can increase production costs

Expanding the use of renewable energy may increase production costs, which depends on several factors. Firstly, the initial investment required for introducing renewable energy facilities, such as solar photovoltaic systems or wind power generation equipment, is usually high. These investments include equipment purchase, installation, and commissioning costs, which may impose a certain burden on the cash flow of the enterprise. Secondly, there are operation and maintenance costs to consider. The operation and maintenance of renewable energy facilities require regular inspections, equipment maintenance, troubleshooting, and other related costs. These additional costs may increase the production costs of the enterprise. Additionally, the supply of renewable energy is affected by factors such as weather and season, which can cause instability in comparison to traditional energy sources. To guarantee a reliable energy supply, the enterprise may need to install backup energy systems or connect to the grid to compensate for the shortage of renewable energy supply [4]. However, this could lead to an increase in production costs. Additionally, there may be transformation and adjustment costs associated with converting traditional energy facilities to renewable energy facilities. This may involve a series of transformations and adjustments, such as equipment replacement and production process improvement, which increase the transformation costs of the enterprise.

**Fig 1:**
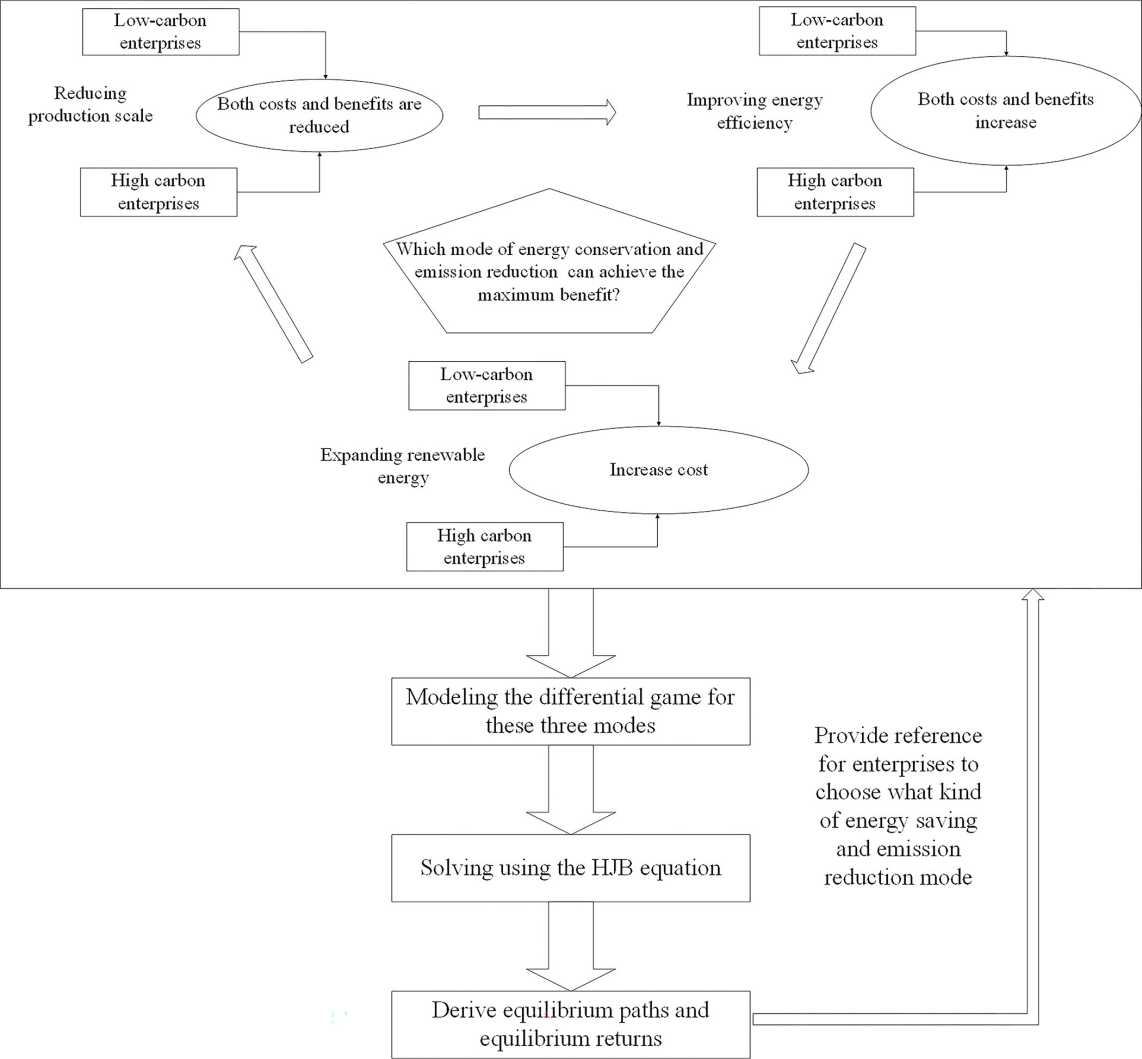
Flowchart of how to select the suitable energy saving and emission reduction mode.

The flowchart of how to select the suitable energy saving and emission reduction mode is shown in Fig 1.

## 3. Differential game model construction

### 3.1 Variable definition

When constructing the differential game model in this article, the author defines many parameters and variables, which are listed in Table 1.

**Table 1:** The main definition of variables and parameters in this article.

variables and parameters	specific meaning
*Y =* {*R*,*I*,*E*}	three modes of controlling heat wave (reduce production scale, improve energy efficiency, expanding renewable energy)
independent variable	
*F*_*Y*1_(*t*)	production of low-carbon enterprises under the control mode *Y*
*F*_*Y*2_(*t*)	production of high-carbon enterprises under the control mode *Y*
*x*_*Y*1_(*t*)	the low-carbon enterprises’ reputation for manufacturing products under the control mode *Y*
*x*_*Y*2_(*t*)	the high-carbon enterprises’ reputation for manufacturing products under the control mode *Y*
parameter	
*ρ*	the discount rate that occurs over time, 0≤*ρ*≤1
*δ*	decay of reputation, *δ*>0
*b*_1_, *b*_2_	the benefits of low-carbon enterprises or high-carbon enterprises in producing units of products, *b*_1_, *b*_2_>0
*c*_1_,*c*_2_	the cost of producing a unit of product by low-carbon enterprises or high-carbon enterprises, *c*_1_, *c*_2_>0
*l*	the positive impact of reputation per unit quantity, *l*>0
*C*_*a*_	carbon emission allowances allocated by the government to low-carbon enterprises, *C*_*a*_>0
*b*_*R*_	Reduced earnings due to reduced scale of production, *b*_*R*_>0
*f*	carbon quota reduction due to reduction in production scale, *f*>0
*c*_*R*_	cost reductions from reduced production scale, *c*_*R*_>0
*b*_*I*_	increased revenue from increasing energy efficiency, *b*_*I*_>0
*c*_*I*_	the added costs of improving energy efficiency, *c*_*I*_>0
*c*_*E*_	the added costs of expanding renewable energy, *c*_*E*_>0
*C*_*I*_	carbon credits generated by increasing energy efficiency, *C*_*I*_>0
*C*_*E*_	expanding carbon credits from renewable energy, *C*_*E*_>0
*h*_1_,*h*_2_	reputation increases for low-carbon or high-carbon enterprises producing units of products, *h*_1_,*h*_2_>0
function	
*J*_*Y*1_(*t*)	the social welfare function of low-carbon enterprises under the control mode *Y*
*J*_*Y*2_(*t*)	the social welfare function of high-carbon enterprises under the control mode *Y*
*V*_*Y*1_(*t*)	the social benefits of low-carbon enterprises under the control mode *Y*
*V*_*Y*2_(*t*)	the social benefits of high-carbon enterprises under the control mode *Y*

In order to make it easier for readers to understand, this paper explains the difficult parameters that appear above.

The discount factor *ρ* is a very important concept in mathematics that is used to calculate the present value or today’s value of a future cash flow. It is based on the fundamental financial principle that a given amount of money has a higher value today than it will have at some point in the future, a phenomenon commonly referred to as "time value" [38]. Similarly, in this paper, the economic or social benefits of energy efficiency and emission reduction can be realized by enterprises, and the discounting of these benefits can be used as a discounting factor. Reputation decay *δ* is the phenomenon whereby the reputation of an individual, company or product declines or deteriorates over time. This decay can be due to a variety of reasons including, but not limited to, negative events, competitive pressures, slow change, lack of communication, and decay of residual influence [39].

The cost *c*_1_,*c*_2_ of producing a unit of product in a company is the average cost that a company spends to produce a single product, which includes direct and indirect costs. First, direct costs. Direct costs are costs directly related to the production of a product. This usually includes the cost of raw materials and direct labor costs. Second, indirect costs. Indirect costs are not directly attributable to the manufacture of a single product, but are costs associated with the entire production process or company operations. This includes manufacturing overhead and administrative overhead [40].

The positive effect *l* of quantity produced per unit on reputation suggests that producing a certain amount of product may have a positive effect on a enterprise’s reputation. These effects include the following main aspects. First, consistency of quality. When a enterprise is able to produce a certain amount of products consistently, this usually means that the enterprise has a mature production process and quality control system. The continuous supply of products of reliable quality enhances consumer trust and therefore has a positive impact on reputation. Second, demand satisfaction. The ability to supply sufficient quantities of products on a consistent basis helps to satisfy market demand by ensuring that customers have timely access to the goods they need, which increases customer satisfaction and their positive opinion of the enterprise. Third, responding to emergency demand [41]. If enterprises are able to quickly increase production quantities to respond to high demand during unexpected events, such as natural disasters or other emergencies, their responsiveness can enhance public perceptions and have a positive impact on enterprises’ reputations.

Government carbon allowances *Ca* for low-carbon enterprises refer to a cap on the amount of carbon emissions allowed to be emitted (i.e., carbon allowances) set by the government based on the objective of reducing greenhouse gas emissions and promoting energy conservation and emission reduction by enterprises. This is usually a policy tool implemented within the framework of a carbon trading market, also known as an "emissions trading system" or "carbon trading system". Under such a system, the government first sets a cap on total carbon emissions and allocates this cap to companies participating in the carbon market. Each enterprise receives a certain number of emission allowances, i.e. the total amount of carbon dioxide (or other greenhouse gases) it is allowed to emit over a certain period of time [42]. For low-carbon businesses, i.e., those that have adopted energy-saving technologies, improved energy efficiency, switched to renewable energy or developed cleaner sources of energy, their carbon emissions are typically lower and they may not use all of the carbon allowances allocated to them.

"Reduced earnings *b*_*R*_ due to reduction in the scale of production" refers to a situation in which an enterprise receives less total revenue because it has reduced the scale of production. The scale of production can be reduced for a variety of reasons, such as a decline in market demand, an increase in the cost of raw materials, technological change, environmental policy constraints, and so on [43]. The reduction in the scale of production can be the result of an active decision or a forced one. In this case, companies may experience the following effects. For example, decline in sales, reduction in market share, weakening of economies of scale, reduction in operating profit, lower return on investment, etc.

"Increased revenues *b*_*I*_ from energy efficiency" refers to situations where a business or organization saves money and/or is able to increase its economic returns as a result of improved efficiency in the use of energy. Improved energy efficiency means that the same or even more products and services are produced with fewer energy resources, which can affect revenues in the following ways. For example, cost savings, cost avoidance, competitive advantage, enhanced brand value, and new revenue streams. Thus, the increased revenues generated through energy efficiency improvements go beyond direct energy cost savings and can affect the financial performance of enterprises through multiple channels [44]. These activities not only drive economic growth, but also benefit the environment, as energy efficiency improvements are often accompanied by reductions in greenhouse gas emissions and other pollutants.

Improving the efficiency of energy use, while reducing energy consumption and costs in the long term, may result in some additional costs *c*_*I*_ in the short term. These additional costs include, among others, initial investment, installation and maintenance costs, training costs, product or service costs, decommissioning and disposal costs, depreciation and opportunity costs. Thus, while improving energy efficiency is often associated with cost savings in long-term considerations, in the short term, enterprises may face additional costs as a result of improvement measures [45]. Nonetheless, such an investment will often pay for itself over time by reducing enough operating costs and improving efficiency. When making such investments, it is important to carry out a thorough cost-benefit analysis to ensure that energy efficiency measures ultimately result in a positive financial return.

There are a range of costs associated with expanding renewable energy programs and resource use. These costs may include initial construction and facility investment costs, land and environmental impact costs, maintenance and operating costs, storage and transmission capacity building costs, technology research and development costs, policy and legal compliance costs, and market competition and price volatility risks. While renewable energy projects may have high start-up and operating costs, their long-term benefits typically include energy price stability, reduced dependence on fossil fuels, lower long-term operating costs, and reduced greenhouse gas emissions [46]. As technology advances and economies of scale are realized, the cost of renewable energy technologies continues to fall, making an increasing number of renewable energy projects economically viable. Government subsidies and incentives (e.g., tax credits, green credits or feed-in tariffs) can also reduce the financial burden on investors and accelerate the payback cycle of these projects.

### 3.2 Differential game of three control modes

Differential games, mathematical models in game theory, describe how multiple players make decisions over continuous time. Unlike discrete-time game models, differential games depict players’ strategies as continuous functions changing over time. Commonly applied in dynamic systems, economics, engineering, and biology, these models have wide-ranging uses. Each player in differential games aims to maximize their utility function by choosing the right strategy. Strategies evolve as players adapt to the current situation and opponents’ strategies. Differential equations describe the continuous evolution of players’ strategies. Studying differential games reveals optimal strategies and equilibria through equation solving, extreme value calculation, and stability analysis. These models help understand game behavior among interacting decision-makers, offering predictions and analyses of rational behavior. Currently, differential games are primarily applied in the fields of supply chain [47], emission reduction [48], and public health [49].

If low-carbon enterprises and high-carbon enterprises adopt the mode of reducing production scale to reduce energy consumption and emission, then the social welfare functions of low-carbon enterprises and high-carbon enterprises are:

$$J_{R1}=\int_{0}^{\infty }[(b_{1}-b_{R})F_{R1}(t)-\frac{c_{1}-c_{R}}{2}F_{R1}^{2}(t)+(C_{a}-fb_{R})+lx_{R1}(t)]e^{-\rho t}dt$$ (1)

$$J_{R2}=\int_{0}^{\infty }[(b_{2}-b_{R})F_{R2}(t)-\frac{c_{2}-c_{R}}{2}F_{R2}^{2}(t)-(C_{a}-fb_{R})+lx_{R2}(t)]e^{-\rho t}dt$$ (2)

In the above formula, (*b*_1_−*b*_*R*_)*F*_*R*1_(*t*) denotes the revenue generated by low-carbon enterprises from product sales. $\frac{c_{1}-c_{R}}{2}F_{R1}^{2}(t)$ represents the production costs of low-carbon enterprises. (*b*_2_−*b*_*R*_)*F*_*R*2_(*t*) denotes the revenue of high-carbon enterprises from their products. $\frac{c_{2}-c_{R}}{2}F_{R2}^{2}(t)$ indicates the production costs of high-carbon enterprises. (*C*_*a*_−*fb*_*R*_) symbolizes the carbon emission allowances acquired by low-carbon enterprises or those that need to be purchased by high-carbon enterprises. For low-carbon enterprises, *fb*_*R*_ reflects the reduction in carbon quotas achieved by scaling down production. For high-carbon enterprises, *fb*_*R*_ signifies the decreased carbon purchase costs when production is scaled down. $\frac{c_{R}}{2}F_{R1}^{2}(t)$ indicates the total cost savings from scaling down production. *lx*_*R*1_(*t*) reflects the beneficial reputation effects on low-carbon enterprises. *lx*_*R*2_(*t*) denotes the positive influence of reputation on high-carbon enterprises.

If low-carbon enterprises and high-carbon enterprises adopt the mode of reducing production scale to reduce energy consumption and emission, then their social welfare functions in this mode can be expressed as:

$${\dot{x}}_{R1}(t)=h_{1}F_{R1}(t)-\delta x_{R1}(t)$$ (3)

$${\dot{x}}_{R2}(t)=h_{2}F_{R2}(t)-\delta x_{R2}(t)$$ (4)

In the above formula, *h*_1_*F*_*R*1_(*t*) represents the reputation of low-carbon enterprises for producing products. *δx*_*R*1_(*t*) denotes the reputation decline in low-carbon enterprises. *h*_2_*F*_*R*2_(*t*) symbolizes the reputation of high-carbon enterprises in product production. *δx*_*R*2_(*t*) reflects the reputation decline of high-carbon enterprises.

If low-carbon enterprises and high-carbon enterprises adopt the mode of improving energy efficiency to reduce energy consumption and emission, then the social welfare functions of low-carbon enterprises and high-carbon enterprises are:

$$J_{I1}=\int_{0}^{\infty }[(b_{1}+b_{I})F_{I1}(t)-\frac{c_{1}+c_{I}}{2}F_{I1}^{2}(t)+C_{a}+C_{I}+lx_{I1}(t)]e^{-\rho t}dt$$ (5)

$$J_{I2}=\int_{0}^{\infty }[(b_{2}+b_{I})F_{I2}(t)-\frac{c_{2}+c_{I}}{2}F_{I2}^{2}(t)-C_{a}+C_{I}+lx_{I2}(t)]e^{-\rho t}dt$$ (6)

In the above formula, (*b*_1_−*b*_*I*_)*F*_*I*1_(*t*) represents the benefits low-carbon enterprises gain from product manufacturing. $\frac{c_{1}+c_{I}}{2}F_{I1}^{2}(t)$ denotes the production costs incurred by low-carbon enterprises. (*b*_2_+*b*_*I*_)*F*_*I*2_(*t*) reflects the benefits gained by high-carbon enterprises from product manufacturing. $\frac{c_{2}+c_{I}}{2}F_{I2}^{2}(t)$ indicates the production costs of high-carbon enterprises. *b*_*I*_*F*_*I*1_(*t*) signifies the additional benefit from enhanced energy efficiency. $\frac{c_{I}}{2}F_{I1}^{2}(t)$ denotes the extra costs associated with enhancing energy efficiency. *C*_*a*_ symbolizes the carbon emission rights awarded to low-carbon companies or required for purchase by high-carbon companies. *lx*_*I*1_(*t*) reflects the positive reputation impact on low-carbon enterprises. *lx*_*I*2_(*t*) denotes the favorable reputation influence on the high-carbon enterprises.

It is noteworthy that energy-saving products have become increasingly popular for their ability to help consumers reduce energy consumption, leading to a rise in market demand for these products. As the market preference for energy-saving products grows, manufacturers may price these products higher. The reason is that energy-saving products typically utilize more efficient technologies or materials, which tend to have higher production costs. At the same time, consumers are willing to pay extra for the long-term benefits of reduced energy consumption that energy-saving products offer. Therefore, despite the potential for energy efficiency benefits and cost savings, the selling price of energy-saving products is often higher than that of non-energy-saving products. *b*_*I*_*F*_*I*1_(*t*) and *b*_*I*_*F*_*I*2_(*t*) denotes the premium that comes with energy-efficient products. $\frac{c_{I}}{2}F_{I1}^{2}(t)$ represents the increased cost of improving utilization efficiency. If $b_{I}F_{I1}(t)+C_{I}-\frac{c_{I}}{2}F_{I1}^{2}(t)$ and $b_{I}F_{I2}(t)+C_{I}-\frac{c_{I}}{2}F_{I2}^{2}(t)$ are both greater than zer, then this mode can be generated and analyzed in comparison with the other two modes. If $b_{I}F_{I1}(t)+C_{I}-\frac{c_{I}}{2}F_{I1}^{2}(t)$ or $b_{I}F_{I2}(t)+C_{I}-\frac{c_{I}}{2}F_{I2}^{2}(t)$ or is less than zero, then the pattern is not valid.

If low-carbon enterprises and high-carbon enterprises adopt the mode of improving energy efficiency to reduce energy consumption and emission, then their social welfare functions in this mode can be expressed as:

$${\dot{x}}_{I1}(t)=h_{1}F_{I1}(t)-\delta x_{I1}(t)$$ (7)

$${\dot{x}}_{I2}(t)=h_{2}F_{I2}(t)-\delta x_{I2}(t)$$ (8)

In the above formula, *h*_1_*F*_*I*1_(*t*) denotes the reputation of low-carbon enterprises in product manufacturing. *δx*_*I*1_(*t*) indicates the decline of the reputation of low-carbon enterprises. *h*_2_*F*_*I*2_(*t*) signifies the reputation of high-carbon enterprises for producing products. *δx*_*I*2_(*t*) reflects the decline of the reputation of high-carbon enterprises.

If low-carbon enterprises and high-carbon enterprises adopt the mode of expanding renewable energy to reduce energy consumption and emission, then the social welfare functions of low-carbon enterprises and high-carbon enterprises are:

$$J_{E1}=\int_{0}^{\infty }[b_{1}F_{E1}(t)-\frac{c_{1}+c_{E}}{2}F_{E1}^{2}(t)+C_{a}+C_{E}+lx_{E1}(t)]e^{-\rho t}dt$$ (9)

$$J_{E2}=\int_{0}^{\infty }[b_{2}F_{E2}(t)-\frac{c_{2}+c_{E}}{2}F_{E2}^{2}(t)-C_{a}+C_{E}+lx_{E2}(t)]e^{-\rho t}dt$$ (10)

In the above formula, *b*_1_*F*_*E*1_(*t*) denotes the revenue low-carbon enterprises earn from product manufacturing. $\frac{c_{1}+c_{E}}{2}F_{E1}^{2}(t)$ indicates the manufacturing costs incurred by the low-carbon enterprises. *b*_2_*F*_*E*2_(*t*) signifies the revenue generated by the high-carbon enterprises through product manufacturing. $\frac{c_{2}+c_{E}}{2}F_{E2}^{2}(t)$ reflects the production costs of the high-carbon enterprises. $\frac{c_{E}}{2}F_{E1}^{2}(t)$ denotes the extra costs incurred by the low-carbon enterprises in expanding renewable energy. $\frac{c_{E}}{2}F_{E2}^{2}(t)$ indicates the additional expenses for the high-carbon enterprises to expand renewable energy. *C*_*a*_ symbolizes the carbon emission rights acquired by the low-carbon enterprises or those required for purchase by the high-carbon enterprises. *lx*_*E*1_(*t*) reflects the beneficial reputation impact on the low-carbon enterprises. *lx*_*E*2_(*t*) signifies the positive effect of reputation on the high-carbon enterprises.

If low-carbon enterprises and high-carbon enterprises adopt the mode of expanding renewable energy to reduce energy consumption and emission, then their social welfare functions in this mode can be expressed as:

$${\dot{x}}_{E1}(t)=h_{1}F_{E1}(t)-\delta x_{E1}(t)$$ (11)

$${\dot{x}}_{E2}(t)=h_{2}F_{E2}(t)-\delta x_{E2}(t)$$ (12)

In the above formula, *h*_1_*F*_*E*1_(*t*) denotes the reputation of low-carbon enterprises for producing goods. *δx*_*E*1_(*t*) indicates the decline of the reputation of low-carbon enterprises. *h*_2_*F*_*E*2_(*t*) symbolizes the reputation of high-carbon enterprises for producing goods. *δx*_*E*2_(*t*) reflects the decline of the reputation of high-carbon enterprises.

## 4. Equilibrium analysis of Differential game

### 4.1 HJB formula

In the differential game, the decisions of low-carbon and high-carbon enterprises on energy saving and emission reduction are influenced by control variables, parameters, and time. To accurately calculate control quantities and social benefits, the HJB formula is employed. The HJB formula, a partial differential equation, constitutes the core of optimal control.

Under the mode of reducing production scale, the HJB equation of the social welfare function of low-carbon enterprises and high-carbon enterprises are:

$$\rho V_{R1}=\underset{F_{R1}(t)}{\max}\{[(b_{1}-b_{R})F_{R1}(t)-\frac{c_{1}-c_{R}}{2}F_{R1}^{2}(t)+(C_{a}-fb_{R})+lx_{R1}(t)]+\frac{\partial V_{R1}}{\partial x_{R1}}[h_{1}F_{R1}(t)-\delta x_{R1}(t)]\}$$ (13)

$$\rho V_{R2}=\underset{F_{R2}(t)}{\max}\{[(b_{2}-b_{R})F_{R2}(t)-\frac{c_{2}-c_{R}}{2}F_{R2}^{2}(t)-(C_{a}-fb_{R})+lx_{R2}(t)]+\frac{\partial V_{R2}}{\partial x_{R2}}[h_{2}F_{R2}(t)-\delta x_{R2}(t)]\}$$ (14)

Under the mode of improving energy efficiency, the HJB equation of the social welfare function of low-carbon enterprises and high-carbon enterprises are:

$$\rho V_{I1}=\underset{F_{I1}(t)}{\max}\{[(b_{1}+b_{I})F_{I1}(t)-\frac{c_{1}+c_{I}}{2}F_{I1}^{2}(t)+C_{a}+C_{I}+lx_{I1}(t)]+\frac{\partial V_{I1}}{\partial x_{I1}}[h_{1}F_{I1}(t)-\delta x_{I1}(t)]\}$$ (15)

$$\rho V_{I2}=\underset{F_{I2}(t)}{\max}\{[(b_{2}+b_{I})F_{I2}(t)-\frac{c_{2}+c_{I}}{2}F_{I2}^{2}(t)-C_{a}+C_{I}+lx_{I2}(t)]+\frac{\partial V_{I2}}{\partial x_{I2}}[h_{2}F_{I2}(t)-\delta x_{I2}(t)]\}$$ (16)

Under the mode of expanding renewable energy, the HJB equation of the social welfare function of low-carbon enterprises and high-carbon enterprises are:

$$\rho V_{E1}=\underset{F_{E1}(t)}{\max}\{[b_{1}F_{E1}(t)-\frac{c_{1}+c_{E}}{2}F_{E1}^{2}(t)+C_{a}+C_{E}+lx_{E1}(t)]+\frac{\partial V_{E1}}{\partial x_{E1}}[h_{1}F_{E1}(t)-\delta x_{E1}(t)]\}$$ (17)

$$\rho V_{E2}=\underset{F_{E2}(t)}{\max}\{[b_{2}F_{E2}(t)-\frac{c_{2}+c_{E}}{2}F_{E2}^{2}(t)+C_{a}+C_{E}+lx_{E2}(t)]+\frac{\partial V_{E2}}{\partial x_{E2}}[h_{2}F_{E2}(t)-\delta x_{E2}(t)]\}$$ (18)

### 4.2 Result of equilibrium

Proposition 1: Under the mode of reducing production scale, the production and social benefits of the low-carbon enterprises and high-carbon enterprises are respectively (the specific solving procedure is shown in S1 Appendix):

$$F_{R1}^{*}(t)=\frac{b_{1}-b_{R}+\frac{l}{\rho +\delta }h_{1}}{c_{1}-c_{R}}$$ (19)

$$F_{R2}^{*}(t)=\frac{b_{2}-b_{R}+\frac{l}{\rho +\delta }h_{1}}{c_{2}-c_{R}}$$ (20)

$$\begin{array}{l} V_{R1}^{*}=\frac{l}{\rho +\delta }x_{R1}+\frac{1}{\rho }(b_{1}-b_{R})\frac{b_{1}-b_{R}+\frac{l}{\rho +\delta }h_{1}}{c_{1}-c_{R}}-\frac{c_{1}-c_{R}}{2}\frac{1}{\rho }{\left(\frac{b_{1}-b_{R}+\frac{l}{\rho +\delta }h_{1}}{c_{1}-c_{R}}\right)}^{2}+\frac{1}{\rho }(C_{a}-fb_{R})\\ \;+\frac{1}{\rho }\frac{l}{\rho +\delta }h_{1}\frac{b_{1}-b_{R}+\frac{l}{\rho +\delta }h_{1}}{c_{1}-c_{R}} \end{array}$$ (21)

$$\begin{array}{l} V_{R2}^{*}=\frac{l}{\rho +\delta }x_{R2}+\frac{1}{\rho }(b_{2}-b_{R})\frac{b_{2}-b_{R}+\frac{l}{\rho +\delta }h_{1}}{c_{2}-c_{R}}-\frac{1}{\rho }\frac{c_{2}-c_{R}}{2}{\left(\frac{b_{2}-b_{R}+\frac{l}{\rho +\delta }h_{1}}{c_{2}-c_{R}}\right)}^{2}-\frac{1}{\rho }(C_{a}-fb_{R})\\ \;+\frac{1}{\rho }\frac{l}{\rho +\delta }h_{2}\frac{b_{2}-b_{R}+\frac{l}{\rho +\delta }h_{1}}{c_{2}-c_{R}} \end{array}$$ (22)

Conclusion 1: The greater the reduction in profits from production scale reduction, the smaller the production volume of the enterprises.

Proposition 2: Under the mode of improving energy efficiency, the production and social benefits of the low-carbon enterprises and high-carbon enterprises are respectively (the specific solving procedure is shown in S2 Appendix):

$$F_{I1}^{*}(t)=\frac{b_{1}+b_{I}+\frac{l}{\rho +\delta }h_{1}}{c_{1}+c_{I}}$$ (23)

$$F_{I2}^{*}(t)=\frac{b_{2}+b_{I}+\frac{l}{\rho +\delta }h_{1}}{c_{2}+c_{I}}$$ (24)

$$\begin{array}{l} V_{I1}^{*}=\frac{l}{\rho +\delta }x_{I1}+\frac{1}{\rho }(b_{1}+b_{I})\frac{b_{1}+b_{I}+\frac{l}{\rho +\delta }h_{1}}{c_{1}+c_{I}}-\frac{c_{1}+c_{I}}{2}\frac{1}{\rho }{\left(\frac{b_{1}+b_{I}+\frac{l}{\rho +\delta }h_{1}}{c_{1}+c_{I}}\right)}^{2}+\frac{1}{\rho }C_{a}+\frac{1}{\rho }C_{I}\\ \;+\frac{1}{\rho }\frac{l}{\rho +\delta }h_{1}\frac{b_{1}+b_{I}+\frac{l}{\rho +\delta }h_{1}}{c_{1}+c_{I}} \end{array}$$ (25)

$$\begin{array}{l} V_{I2}^{*}=\frac{l}{\rho +\delta }x_{I2}+\frac{1}{\rho }(b_{2}+b_{I})\frac{b_{2}+b_{I}+\frac{l}{\rho +\delta }h_{1}}{c_{2}+c_{I}}-\frac{c_{2}+c_{I}}{2}\frac{1}{\rho }{\left(\frac{b_{2}+b_{I}+\frac{l}{\rho +\delta }h_{1}}{c_{2}+c_{I}}\right)}^{2}-\frac{1}{\rho }C_{a}+\frac{1}{\rho }C_{I}\\ \;+\frac{l}{\rho +\delta }\frac{1}{\rho }h_{2}\frac{b_{2}+b_{I}+\frac{l}{\rho +\delta }h_{1}}{c_{2}+c_{I}} \end{array}$$ (26)

Conclusion 2: The greater the increase in revenue from improving energy efficiency, the greater the production volume of the enterprise.

Proposition 3: Under the mode of expanding renewable energy, the production and social benefits of the low-carbon enterprises and high-carbon enterprises are respectively (the specific solving procedure is shown in S3 Appendix):

$$F_{E1}^{*}(t)=\frac{b_{1}+\frac{l}{\rho +\delta }h_{1}}{c_{1}+c_{E}}$$ (27)

$$F_{E2}^{*}(t)=\frac{b_{2}+\frac{l}{\rho +\delta }h_{2}}{c_{2}+c_{E}}$$ (28)

$$\begin{array}{l} V_{E1}^{*}=\frac{l}{\rho +\delta }x_{E1}+\frac{1}{\rho }b_{1}\frac{b_{1}+\frac{l}{\rho +\delta }h_{1}}{c_{1}+c_{E}}-\frac{c_{1}+c_{E}}{2}\frac{1}{\rho }{\left(\frac{b_{1}+\frac{l}{\rho +\delta }h_{1}}{c_{1}+c_{E}}\right)}^{2}+\frac{1}{\rho }C_{a}+\frac{1}{\rho }C_{E}\\ \;+\frac{l}{\rho +\delta }\frac{1}{\rho }h_{1}\frac{b_{1}+\frac{l}{\rho +\delta }h_{1}}{c_{1}+c_{E}} \end{array}$$ (29)

$$\begin{array}{l} V_{E2}^{*}=\frac{l}{\rho +\delta }x_{E2}+\frac{1}{\rho }b_{2}\frac{b_{2}+\frac{l}{\rho +\delta }h_{2}}{c_{2}+c_{E}}-\frac{c_{2}+c_{E}}{2}\frac{1}{\rho }{\left(\frac{b_{2}+\frac{l}{\rho +\delta }h_{2}}{c_{2}+c_{E}}\right)}^{2}-\frac{1}{\rho }C_{a}+\frac{1}{\rho }C_{E}\\ \;+\frac{l}{\rho +\delta }\frac{1}{\rho }h_{2}\frac{b_{2}+\frac{l}{\rho +\delta }h_{2}}{c_{2}+c_{E}} \end{array}$$ (30)

Conclusion 3: The greater the increase in costs of expanding renewable energy, the smaller the production volume of enterprises.

## 5. Numerical analysis

### 5.1 Data description

In order to describe in more detail the changes in the social utility of low-carbon and high-carbon enterprises in the process of energy saving and emission reduction, this paper adopts a numerical analysis method.

The discount factor is primarily a reflection of the uncertainty and potential risk of future cash flows. It is a financial term used to discount future cash flows to their present value. It is a mathematical method of assessing the value of an investment, project or any future cash flow. In other words, a discount factor is a factor used to determine the value that money will have in the present after a certain period of time [50]. At the same time, Bai et al. [51] used the differential game model, and they used the discount factor. Based on how Bai et al. [51] set the parameters, this paper assumes that the discount rate *ρ* that occurs over time is 0.9.

The term "decay of reputation" typically indicates a gradual loss of reputation or credibility over time in specific contexts. This decline may result from various factors, including unreliability, reports of misconduct, decreased market competitiveness, or even the impact of being forgotten. It is usually considered to be minor. In this article, it is not the primary parameter under analysis, and its magnitude within a specific range does not influence the study’s outcomes [52]. Thus, for convenience, this article assumes that decay *δ* of reputation is 0.1

Under a carbon trading system, participants—typically industrial enterprises—receive a specific number of carbon emission allowances or must buy them to offset their emissions. A company exceeding its greenhouse gas emissions quota, such as for carbon dioxide, must buy extra allowances. Conversely, if its emissions are below the quota, it may sell or save the surplus allowances. Deciding to downsize production typically leads to lower energy use and reduced emissions. Consequently, this reduces the need for carbon allowances. The business options include selling surplus allowances on the carbon market for profit or retaining them for potential future production increases or stricter emission policies. As participants continue in this scenario, the "benefits of downsizing" will eventually equal the "reduction in carbon allowances" caused by downsizing. Refer to the parameters set by other scholars [53, 54], this paper assumes that benefits *b*_*R*_ from reducing production scale is 1.5. Carbon quota reduction *f* due to reduction in production scale is 1.5.

Both carbon credits from enhanced energy efficiency and those expanded through renewable energy target greenhouse gas emissions reduction. If carbon credits gained through improving energy efficiency exceed those from expanding renewable energy, enterprises will opt to enhance their energy efficiency [32]. This principle also holds true in the reverse scenario. Therefore, in the long term, carbon credits from energy efficiency and from renewable energy expansion are equivalent. Carbon credits *C*_*I*_ generated by increasing energy efficiency is 3. Expanding carbon credits *C*_*E*_ from renewable energy is 3. An otherwise high-emitting enterprise may become a low-carbon enterprise as a result of implementing a large-scale low-carbon technology transition. With the right policy incentives, investment support or technological innovation, the transformation of a high-carbon enterprise into a low-carbon enterprise often requires the implementation of multiple low-carbon projects and a range of strategies [43]. As a result, the carbon credits *Ca* of a low-carbon enterprise are generated by multiple projects and are much larger than the carbon credits *C*_*I*_ or *C*_*E*_ obtained from a single project. Therefore, this paper hypothesizes that carbon emission allowances *Ca* allocated by the government to low-carbon enterprises is 10.

There are reputations under each energy efficiency model, and the impact of reputation is not the focus of this paper. Its value has no impact on this paper. Bai et al. [51] used differential games and did numerical analysis. Based on how Bai et al. [51] set the parameters, this paper assumes that the positive impact *l* of reputation per unit quantity is 1.

The cost of producing a unit by either a low-carbon or high-carbon enterprise is typically analyzed relative to its benefits. Typically, one variable is held constant while the other is varied for analysis. In this paper, the costs are fixed and in the next process this paper will analyze the benefits. Referred to how Liu et al. [42] set the parameters, this paper assumes that the cost *c*_*1*_, *c*_*2*_ of producing a unit of product by low-carbon enterprises or high-carbon enterprises is 3.

Although the reputation growth per unit of product for low-carbon and high-carbon enterprises exists, it is very difficult to measure. However, this is not the focus of the current study. Its size does not affect the results. Referred to how Gao et al. [55] set the parameters, this paper hypothesizes that reputation increases *h*_*1*_,*h*_*2*_ for low-carbon or high-carbon enterprises producing units of products is 2.

This paper focuses on how revenue from the production of a product by low-carbon or high-carbon enterprises impacts social welfare. Therefore, while analyzing a specific benefit, other related benefits should remain constant. Bai and Wang [56] used differential games and did numerical analysis. Based on how they set the parameters, this paper assumes that increased revenue *b*_*I*_ from increasing energy efficiency is 1.

### 5.2 Results and analysis

When the cost reductions *c*_*R*_ from reduced production scale, the added costs *c*_*I*_ of improving energy efficiency, and the added costs *c*_*E*_ of expanding renewable energy is 1, this article can calculate the social benefits of low-carbon enterprises:

$$V_{R1}^{*}=8.78+0.278{\left(b_{1}+0.5\right)}^{2}$$ (31)

$$V_{I1}^{*}=15.44+0.139{\left(b_{1}+3\right)}^{2}$$ (32)

$$V_{E1}^{*}=15.44+0.139{\left(b_{1}+2\right)}^{2}$$ (33)

**Fig 2:**
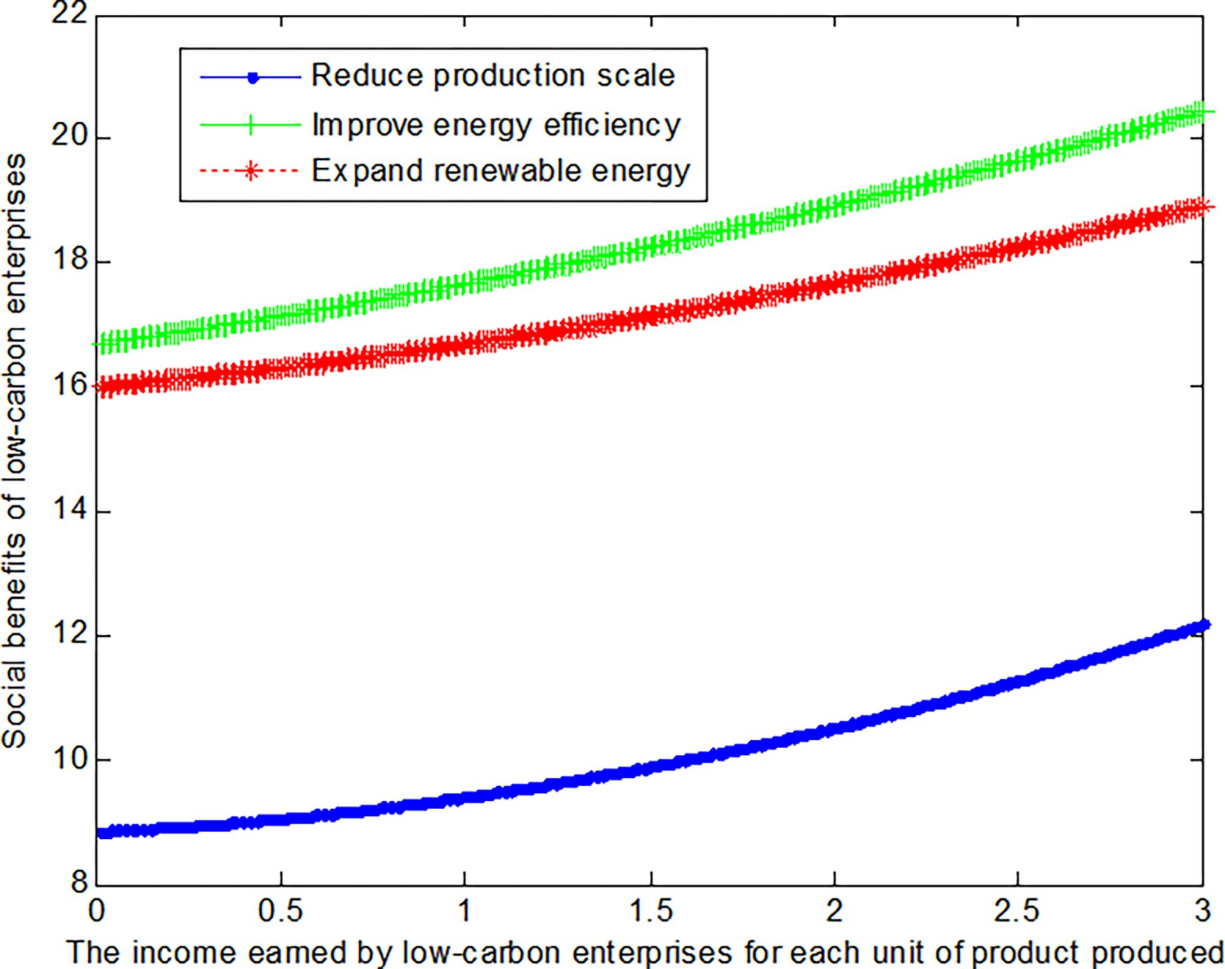
Impact of income of low-carbon enterprises on social welfare.

The following graph (named Fig 2) can also be produced:

Conclusion 4: When the cost of changing the production mode is small, low-carbon enterprises can obtain the maximum benefits by adopting the mode of improving energy efficiency.

When the cost reductions *c*_*R*_ from reduced production scale, the added costs *c*_*I*_ of improving energy efficiency, and the added costs *c*_*E*_ of expanding renewable energy is 2, this article can calculate the social benefits of low-carbon enterprises:

$$V_{R1}^{*}=8.78+0.556{\left(b_{1}+0.5\right)}^{2}$$ (34)

$$V_{I1}^{*}=15.44+0.111{\left(b_{1}+3\right)}^{2}$$ (35)

$$V_{E1}^{*}=15.44+0.111{\left(b_{1}+2\right)}^{2}$$ (36)

**Fig 3:**
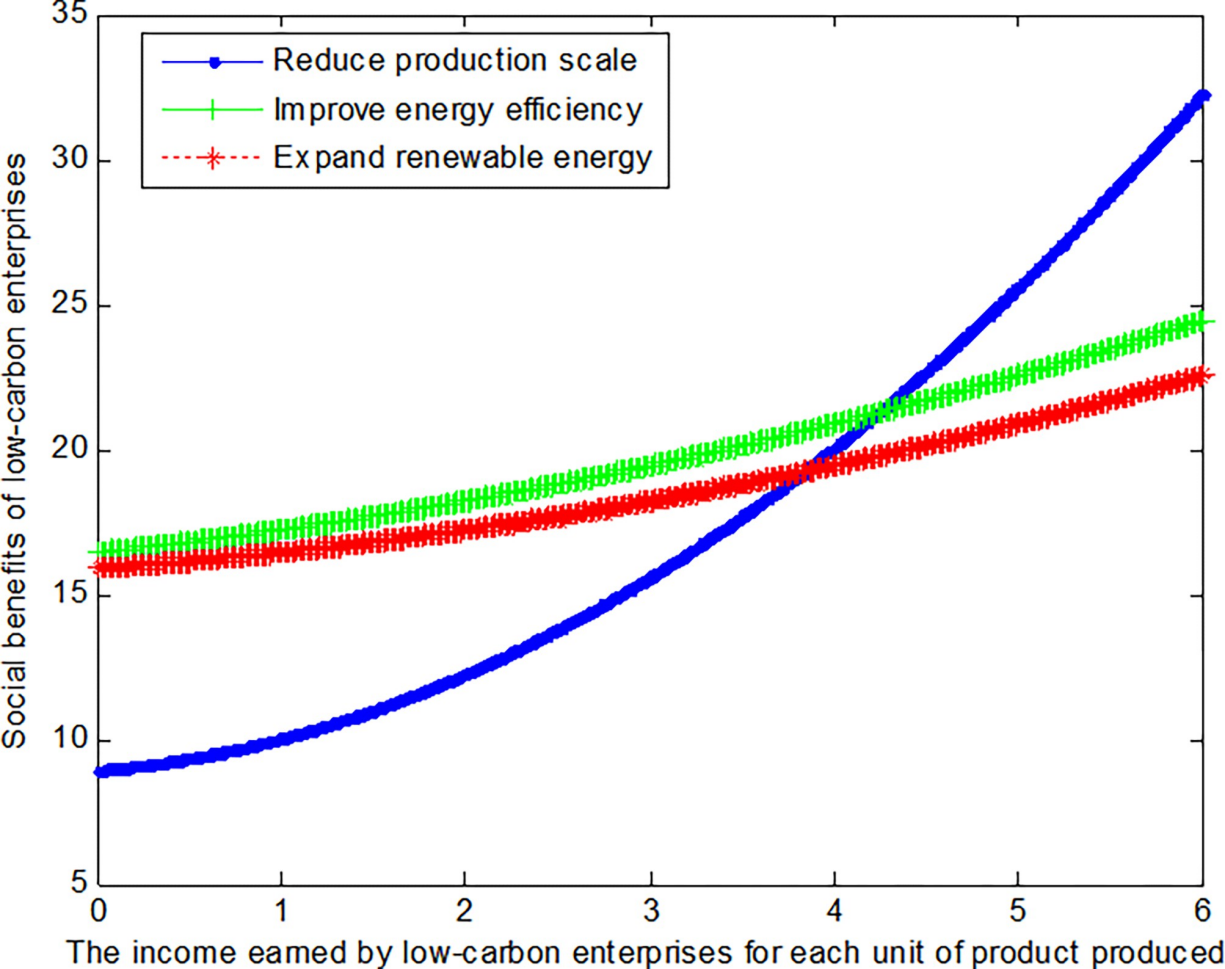
Impact of income of low-carbon enterprises on social welfare.

The following graph (named Fig 3) can also be produced:

Conclusion 5: When the cost of changing the production mode is large and the income obtained from the production of unit product is small, the low-carbon enterprise can obtain the maximum benefit by improving the energy efficiency mode. The mode of improving energy efficiency can increase benefits by about 5.5% over the mode of expanding renewable energy. With the increase of the income obtained from the production of unit product, the low-carbon enterprise can obtain the maximum benefit by reducing the production scale mode. When the cost of changing the production mode and the income obtained from the production of unit product is large, the low-carbon enterprise can obtain the maximum benefit by reducing the production scale mode.

When the cost reductions *c*_*R*_ from reduced production scale, the added costs *c*_*I*_ of improving energy efficiency, and the added costs *c*_*E*_ of expanding renewable energy is 1, this article can calculate the social benefits of high-carbon enterprises:

$$V_{R2}^{*}=-6.78+0.278{\left(b_{2}+0.5\right)}^{2}$$ (37)

$$V_{I2}^{*}=-6.78+0.139{\left(b_{2}+3\right)}^{2}$$ (38)

$$V_{E2}^{*}=-6.78+0.139{\left(b_{2}+2\right)}^{2}$$ (39)

**Fig 4:**
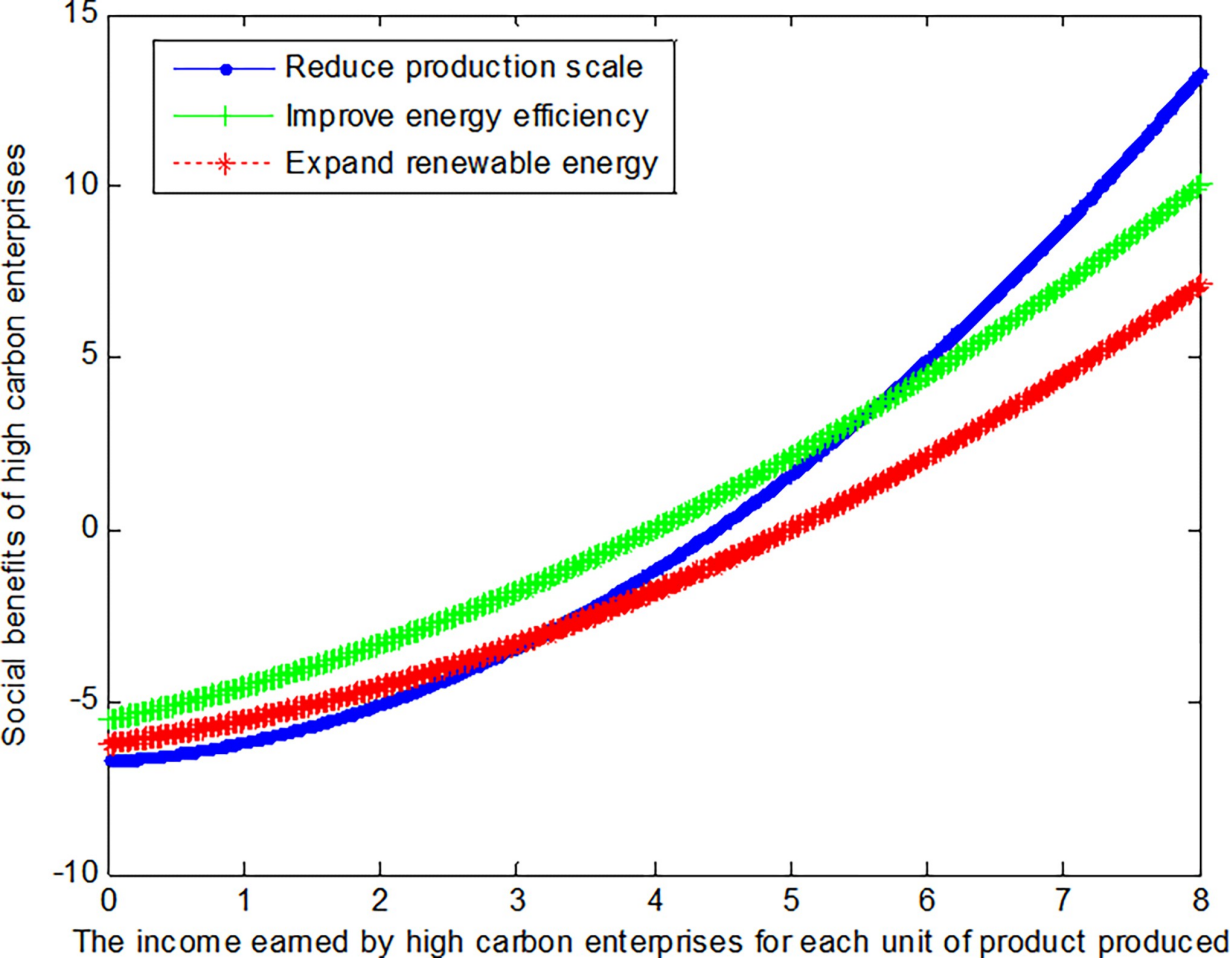
Impact of income of high-carbon enterprises on social welfare.

The following graph (named Fig 4) can also be produced:

When the cost reductions *c*_*R*_ from reduced production scale, the added costs *c*_*I*_ of improving energy efficiency, and the added costs *c*_*E*_ of expanding renewable energy is 2, this article can calculate the social benefits of high-carbon enterprises:

$$V_{R2}^{*}=-6.78+0.556{\left(b_{2}+0.5\right)}^{2}$$ (40)

$$V_{I2}^{*}=-6.78+0.111{\left(b_{2}+3\right)}^{2}$$ (41)

$$V_{E2}^{*}=-6.78+0.111{\left(b_{2}+2\right)}^{2}$$ (42)

**Fig 5:**
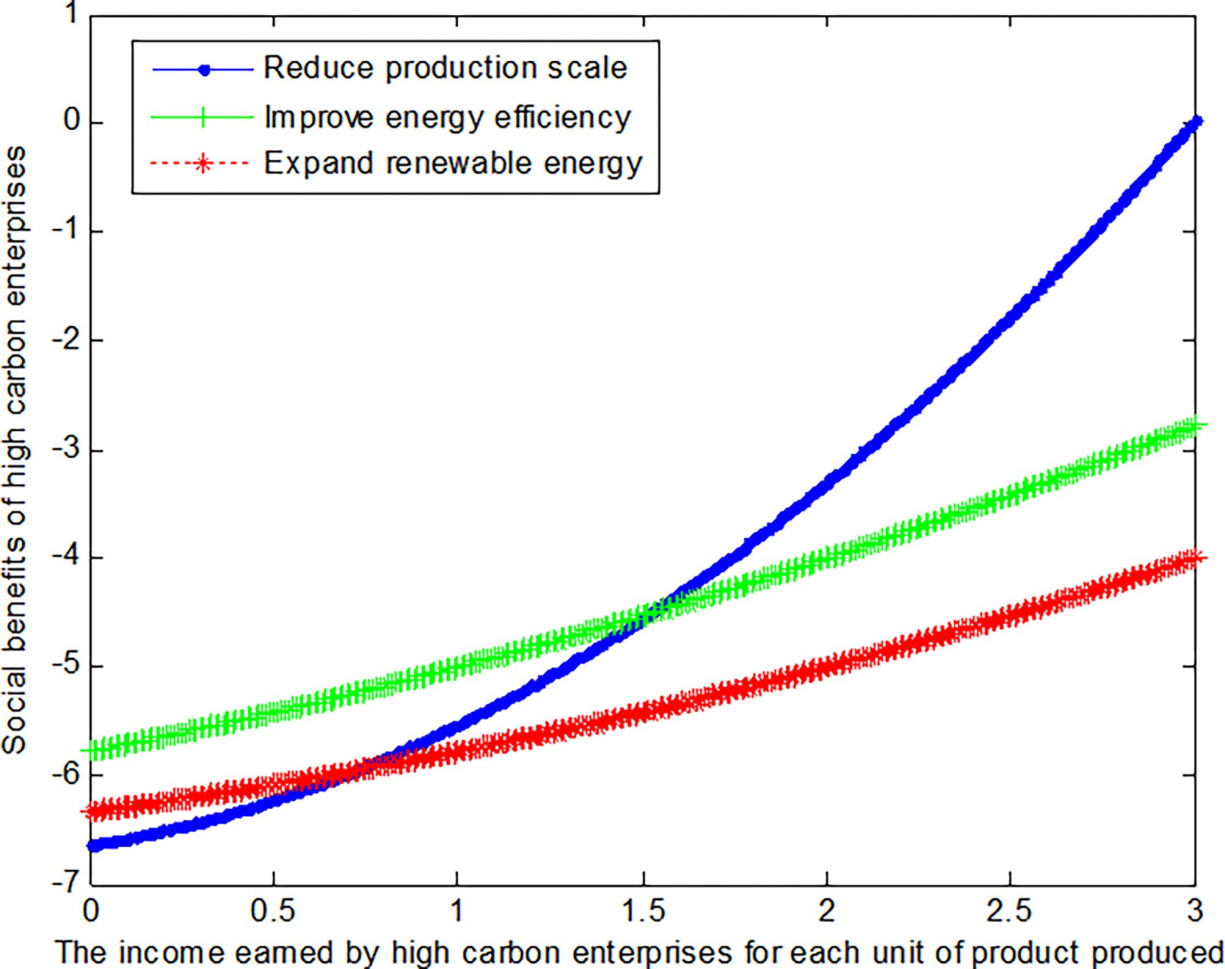
Impact of income of high-carbon enterprises on social welfare.

The following graph (named Fig 5) can also be produced:

Conclusion 6: When the revenue per unit product is small, high-carbon enterprises can obtain the maximum benefit by improving energy efficiency. The mode of improving energy efficiency can increase benefits by about 20.5% over the mode of expanding renewable energy. With the increase of revenue per unit product, high-carbon enterprises can obtain the maximum benefit by reducing production scale. For both low-carbon and high-carbon enterprises, reducing production scale is the fastest way to enhance efficiency when the costs of energy conservation and emission reduction are substantial.

## 6. Discussion

Carbon trading, as a market-based mechanism, permits enterprises to meet national or regional emission reduction targets through the buying and selling of carbon emission rights. Yet, China’s carbon market is currently limited to the power sector. This means that existing market mechanisms are managing and controlling carbon emissions specifically for power generation enterprises [26]. In light of this, the results of this study could be particularly instructive for the power industry, as it directly participates in the existing carbon trading market in China. These findings may assist power companies in assessing and selecting the most effective emission reduction strategies to optimize their performance in the carbon market, thereby achieving the most cost-effective energy conservation and emissions reduction. However, the direct extrapolation of these research outcomes to all enterprises ought to be considered cautiously. While other industries may not directly engage in the carbon trading market at present, their emission reduction strategies and targets could be indirectly influenced by carbon pricing and policy frameworks. Furthermore, there is considerable variance amongst industries in terms of their characteristics, energy consumption structures, and potential for emissions reduction, necessitating tailored emission reduction strategies based on their specific circumstances. In sum, although this study is focused on energy-saving and emission reduction strategies under carbon trading, with power companies as its research subject, its results are directly applicable to enterprises within the power industry. For companies in other industries, although these outcomes can provide certain references and insights, the application of these strategies must still consider the unique conditions and influential factors specific to their industry to make the most suitable emission reduction decisions.

The dynamic between low-carbon and high-carbon enterprises, within the context of energy conservation and emission reduction, holds significant theoretical and empirical value. The specific theoretical significance can be expressed in the following aspects. First, the theoretical model offers insights into scarce natural resources and environmental capacity, and enterprises’ strategic decision-making within these constraints. Second, game theory presents a framework for examining firm behavior and interactions, focusing on both competitive and cooperative dynamics. Various game models elucidate enterprises’ strategic choices and interactions. Third, low-carbon economy models prioritize minimizing environmental costs while maximizing economic benefits. Theoretical analysis uncovers how different strategies affect costs and benefits. Fourth, grasping enterprises’ responses to government policies aids in crafting effective incentives and policy tools, like carbon taxes, trade regimes, and subsidies. Specific practical implications can be expressed in the following ways. First, comparing theoretical model predictions with actual enterprise behavior and outputs assesses environmental policies’ effectiveness and their impact. Second, the study identifies how low-carbon technologies are applied and promoted, and how technological innovation influences emission reduction efficiency and market competition. Third, the behavior of low-carbon and high-carbon enterprises influences market trends, like energy prices and carbon emission rights, assessing firms’ strategic transformation impact. In conclusion, the interplay between low-carbon and high-carbon enterprises offer a critical lens for evaluating energy conservation and emission reduction policies, with the blend of theoretical and empirical analysis serving as a vital guide for shaping environmental policies.

According to Conclusion 1, the smaller the production volume of the enterprise is, the greater the reduction in revenue caused by the reduction in production scale. This conclusion is consistent with the behavior of enterprises in reality to a certain extent. Small enterprises, due to their limited size, market influence, and weaker capabilities in resource and capital mobility, are likely to be more sensitive and severely affected by any changes in production scale than larger enterprises. Several important insights can be drawn from this research result. First, small enterprises should strive to enhance their operational flexibility to quickly adjust production scale and minimize losses when facing reduced demand or other external negative impacts [57]. Second, small enterprises relying on a single product or service are more susceptible to market fluctuations. Therefore, such enterprises should consider diversifying their products or services to spread risk. Third, maintaining good relationships with existing customers is particularly important when production scale needs to be reduced. Stable customer relationships can somewhat cushion the impact of production adjustments. These insights are not only applicable to small enterprises but also offer valuable references for enterprises of all sizes, especially in the context of current economic globalization and intense market competition. Enterprises need to continuously optimize their internal operations and remain sensitive and adaptable to external changes to maintain their market position and economic efficiency. This article diverges from Yadolladi and Matin’s [58] research, which posits that centralized resource allocation reduces the scale in a two-stage network production system; however, both studies focus on production scale reduction. This article examines the effects of reducing production scale. The primary reason, as outlined in Conclusion 1, is that this reduction lowers revenue due to decreased sales volume and profit. This occurs because a smaller production scale restricts the number of products an enterprise can produce and sell. Reduced production volume can decrease revenue in several ways. Firstly, a decrease in sales volume directly affects the enterprises’ operating income. Lower sales volume may reduce the enterprises’ profitability and liquidity. Secondly, a reduced production scale could lead to decreased production efficiency. A smaller production scale could restrict the use of the enterprise’s capacity and resources, reducing both efficiency and cost-effectiveness per unit of product. Consequently, this can raise the enterprise’s costs and adversely affect its profitability.

According to Conclusion 2, improving energy efficiency leads to an increase in production volume for an enterprise. This is generally consistent with the behavior of enterprises in reality. In the field of business management, energy efficiency is regarded as one of the key factors in reducing costs and enhancing production efficiency. By improving energy efficiency, enterprises can not only reduce the cost of energy consumption but also enhance the efficiency of the production process, potentially leading to an increase in production volume. This research result provides several implications for enterprises. First, cost savings and environmental responsibility. Improving energy efficiency helps reduce production costs and lessen the impact on the environment, which is particularly important in the context of global climate change and challenges of environmental sustainability [59]. Second, long-term competitive advantage. By enhancing energy efficiency and incorporating it as part of their business strategy, enterprises can establish a long-term competitive advantage in the market. This is because efficient energy use can improve the overall operational efficiency and cost-effectiveness of a company. Third, stimulating innovation. The pursuit of energy efficiency can inspire enterprises to innovate technologically and optimize processes, such as developing more efficient production technologies and adopting advanced energy management systems. In summary, improving energy efficiency undoubtedly provides enterprises with an opportunity to increase production volume, reduce costs, and enhance market competitiveness. This requires enterprise leadership to pay more attention to energy efficiency and consider it an important part of their business development strategy. This article analyzes the impact of energy efficiency on production volume. This article differs from Huang et al.’s [37] research conclusions, which stated that low comprehensive efficiency was mainly due to insufficient pure technical efficiency. Improving energy efficiency offers several benefits such as cost savings, resource protection, and environmental advantages, which support Conclusion 2. Such benefits could positively influence an enterprises’ production volume. First, enhancing energy efficiency can lower an enterprises’ energy consumption costs. Adopting more efficient equipment and processes can decrease an enterprise’s energy demand and costs. Improving energy efficiency can enhance an enterprise’s competitiveness by reducing production costs and resource consumption. Consequently, this can prolong resources’ lifespan, reduce the demand for natural resources, and yield economic and sustainable gains. By minimizing resource waste, enterprises can improve efficiency and ensure a stable supply during resource shortages. At the same time, Conclusion 2 is validated by real-world examples and data. For example, the African continent currently needs up to $2.8 trillion to implement its Nationally Owned Contributions (NDCs) between 2020 and 2030, compared to the $30 billion currently flowing into the continent for climate finance. Due to the large incremental costs of scaling up renewable energy, the Alliance has seen a 150% increase in members developing resilience plans, with 80% of them now explicitly considering climate risks and reducing production [60]. To summarize, enhancing energy efficiency can provide numerous economic and environmental advantages, motivating enterprises to increase production. By decreasing energy expenses, conserving resources, and meeting environmental standards, enterprises can enhance their competitiveness and sustainability, resulting in increased production.

Conclusion 3 states that as the costs of expanding renewable energy increase, the production volume of the enterprise decreases. This research result aligns with the behavior of enterprises under certain circumstances but involves consideration of various factors. Initially, the investment cost of renewable energy technologies is usually high, which may impact the short-term financial situation of companies, especially those with limited cash flow or shorter expectations for energy investment returns. However, in the long term, as the costs of renewable energy technologies decrease and their efficiency improves, the overall cost of adopting renewable energy technologies for enterprises will reduce. The savings on energy costs could offset the initial investment costs and even enhance production efficiency and volume. The implications of this research result for enterprises include: Firstly, long-term planning. Enterprises should focus on the long-term savings in energy costs and the potential benefits of renewable energy technologies, rather than merely on short-term investment returns. Secondly, risk management. With the global increase in awareness of sustainable development and environmental protection, companies relying on traditional energy sources may face higher policy and market risks [61]. Therefore, enterprises need to balance the long-term benefits of investing in renewable energy against the short-term costs as part of their risk management strategy. Thirdly, leveraging technological advancements. Companies should closely monitor innovations and progress in the field of renewable energy technologies, using these advancements to reduce costs and improve energy efficiency. In summary, although the expansion costs of renewable energy may impose economic pressures on enterprises in the short term, leading to a decrease in production volume, from a long-term perspective, investment in renewable energy not only benefits environmental sustainability but can also bring economic benefits and competitive advantages in the market to enterprises. Therefore, enterprises should adopt a strategic perspective, comprehensively considering the long-term value of investments in renewable energy. This contrasts with Meng et al.’s [62] proposed calculation framework, which more comprehensively and accurately reflects the costs of the deep peak regulation process of coal-fired units. This framework aids in establishing benchmarks and setting fair compensation within the peak regulation auxiliary service market. The study examines how the costs of renewable energy influence production volumes. The study by Meng et al. [62] assists enterprises in mitigating cost increases due to renewable energy adoption. Conclusion 3 attributes the higher costs primarily to the construction and operation of renewable energy facilities. For example, building and maintaining solar and wind power stations entail substantial investment. Enterprises will absorb some of these costs, leading to higher production expenses. However, this increase in costs does not necessarily imply a reduction in production volumes. As renewable energy technology advances and costs decline, many enterprises are adopting it effectively in their production processes. The use of renewable energy offers several benefits including reduced dependency on fossil fuels, decreased environmental pollution, and lessened climate change impacts. Conclusion 3 is supported by real-life cases and data. For example, Holaluz-Clidom SA is a Barcelona-based energy transition company specializing in energy management systems as well as rooftop solar panel installation. This unique combination of products and services contributes to Holaluz’s vision of transforming the current model of energy production (centralized and non-renewable) into a new model based on distributing 100% green energy. Since its inception in 2010, the company has realized the potential of more than 12,000 rooftops and has managed to avoid 2.3 megatons of carbon emissions. Holaluz has created and manages a network of home energy systems that can take advantage of the green energy surplus of its solar installations. The aim is to democratize access to clean and zero-kilometer energy by using neighborhood networks. Holaluz’s ESG risk rating has increased from 12 to 11.6 in 2022 [63]. The company maximizes its positive impact on the planet and people through a model of energy efficiency.

According to Conclusion 4, low-carbon enterprises can achieve maximum benefits by adopting the mode of improving energy efficiency when the cost of changing the production mode is small. This indeed aligns with corporate behaviors observed in reality, especially against the backdrop of enhanced environmental awareness and the drive for sustainability. From a practical standpoint of business operations, many companies, particularly those committed to reducing their carbon footprint, have discovered that improving energy efficiency is often the most cost-effective and immediately impactful way to transform production modes. The implications for businesses include, but are not limited to, the following aspects. Firstly, cost-effectiveness. Research emphasizes the importance of cost efficiency in transitioning towards low-carbon operations, recommending that businesses prioritize changes that can significantly enhance energy efficiency at minimal costs [64]. This approach can quickly yield success through reduced energy bills and bolstered overall sustainability of the enterprise. Secondly, strategic investment. Companies should evaluate how initial investments in energy efficiency improvements could result in long-term savings and environmental benefits. This strategic focus on high-efficiency technologies can better position a company within competitive markets, especially as consumers increasingly prefer environmentally friendly businesses. Thirdly, incremental changes. The findings underscore the advantages of progressive changes as opposed to costly comprehensive overhauls. By gradually increasing energy efficiency, businesses can effectively manage costs while continuing to advance towards broader environmental goals. In summary, although the initial costs of modifying production models towards low-carbon operations might pose a hurdle, when such changes are strategically implemented with an emphasis on enhancing energy efficiency within controllable costs, businesses not only adhere to sustainable development practices but also achieve considerable cost savings and competitive advantages. This research outcome offers encouragement for companies seeking to progress towards greater sustainability without the need for substantial upfront investments. Although both studies examine the impact of cost on companies, this article contradicts the research results of Ding et al. [65]. Ding et al. [65] argue that administrative penalties can raise debt expenses. However, this article analyzes how changes in production costs affect mode selection. When it’s cost-effective to change production modes, low-carbon enterprises benefit most from enhancing energy efficiency. This is due to energy efficiency improvements reducing consumption and, thus, the enterprises’ energy costs. Boosting energy efficiency controls costs and enhances profit margins since energy expenses are among the primary costs in production. Meanwhile, actual cases and data support Conclusion 4. For example, Morningstar Sustainalytics scored six companies, PepsiCo, McDonald’s, Starbucks, Tyson Foods, and Chipotle Mexican Grill Inc. in the United States, as well as a Brazilian company, JBS (Jose Batista Sobrinho), on their low-carbon transition. Their management scores are 70.8, 61, 55.7, 53.4, 52.9, 52. The more they score, the better. The implied warming from their products is 3.4°C, 5.5°C, 4.2°C, 3.1°C, 4.6°C, 4.5°C [66]. Their goal is to reduce implied warming to 1.5°C. While many large food companies have committed to net-zero emissions by 2050, the data shows that they are lagging far behind. Even PepsiCo, which appears to be setting the benchmark for the industry, has significant blind spots in its approach and therefore faces significant transition risks and challenges. If the company’s management policies are not enough, the company’s board of directors and management team must also consider other, more drastic improvements in energy efficiency to achieve emissions reductions. The use of low carbon fuels such as biogas or hydrogen from its own value chain may be required to reduce emissions [66]. In summary, low-carbon enterprises can reduce costs, minimize environmental impacts, and maximize benefits by improving energy efficiency when the cost of changing production mode is relatively low.

According to Conclusion 5, low-carbon enterprises can achieve maximum benefit by improving energy efficiency when the cost of changing production mode is high and revenue obtained from the production unit is low. As revenue increases, the maximum benefit can be achieved by reducing production scale. This indeed reflects certain strategic trends in business behaviors in the real world, especially in industries actively pursuing sustainability. In situations where the cost of transforming production practices becomes prohibitive, focusing on enhancing energy efficiency offers a feasible and cost-effective pathway for low-carbon enterprises looking to optimize operations without significant financial investment. The implications of this research for businesses are multifaceted. Firstly, it underscores the importance of prioritizing energy efficiency improvements when faced with financial constraints [67]. This suggests that initially, conducting cost-benefit analyses on investments in energy efficiency is more rational and sustainable than simply increasing production scale or investing in costly technology upgrades. Secondly, the findings indicate that as business revenue increases and market positioning solidifies, gradually adjusting production scale to align with market demand and environmental goals can maximize both economic and environmental benefits. This strategy not only helps reduce the resource wastage and environmental pressure from overproduction but also promotes a vision for long-term sustainable development of enterprises. In summary, this research provides valuable insights for low-carbon enterprises on how to smartly adjust their production modes and scales under limited resources to achieve sustainability objectives, ultimately realizing a win-win situation for both economic benefits and environmental protection. This finding diverges from Zhang et al.’s [68] research. According to Zhang et al. [68], excessive government regulation in the initial stages can diminish the economic benefits of low-carbon sustainable development for enterprises. This article mainly emphasizes cost changes and production efficiency, whereas Zhang et al. [68] focus on enterprise development stages and government regulation levels. When changing production modes is costly and unit revenue is low, low-carbon enterprises can greatly benefit from enhancing energy efficiency. This is due to the fact that a change in production mode can necessitate substantial resources and funds, which are better allocated to improving energy efficiency. In cases of low revenue, low-carbon enterprises can lower energy costs and boost profit margins by enhancing energy efficiency. Concurrently, practical examples and data validate Conclusion 5. For example, 694 kilotons (Kt) of battery waste and 5,108 tons of carbon dioxide equivalent (tCO2e) emissions will be avoided by implementing Cota when a company’s revenues increase. The report estimates that Cota implementation has the potential to prevent the mining of 7.8 tons of lithium and 15.6 million liters of water over the same period [69]. In summary, when the cost of changing the production mode is high and the revenue is low, low-carbon enterprises can reduce costs by improving energy efficiency to achieve maximum benefits. As revenue increases, low-carbon enterprises can further improve revenue by reducing production scale to ensure a balance between supply and demand and optimize resources. The modification of this strategy assists in attaining the optimal business objectives of enterprises in various economic environments.

According to Conclusion 6, high-carbon enterprises can achieve maximum benefit by improving the energy efficiency mode when the revenue per unit product is small. As the revenue per unit product increases, high-carbon enterprises can achieve maximum benefit by reducing the production scale. This indeed aligns with real-world business behaviors. Faced with growing environmental pressures and demands for energy savings and emission reductions, businesses, especially those in high carbon emitting industries, are seeking operational strategies that can satisfy both economic benefits and environmental responsibilities. The implications for businesses are manifold. Firstly, high-carbon enterprises should value the strategic importance of enhancing energy efficiency. Even at stages of lower income, improving energy efficiency can yield significant economic and environmental benefits. The enhancement of energy efficiency not only reduces energy consumption and costs but can also decrease carbon emissions to some extent, positively affecting the market competitiveness and social image of the enterprise. Secondly, timely adjustments to production scale in response to market and environmental changes are necessary. As the revenue per unit product for the enterprise increases, there should be a proper reduction in production scale according to market demand and environmental goals. This can optimize resource allocation, reduce unnecessary production and inventory costs, and help lower the enterprise’s carbon footprint, leading to more sustainable development. Thirdly, the importance of long-term planning and flexibility in responding to changes is paramount. In pursuit of economic benefits, businesses also need to continually examine and adjust their production and operating modes to ensure sustainable development in the changing market and policy environment [70]. This requires businesses to not only focus on current economic indicators but also anticipate future environmental requirements and market demand changes. In summary, this research outcome encourages high-carbon enterprises to actively seek maximization of environmental benefits while pursuing economic gains. By adopting different strategic measures at various stages, enterprises can not only enhance their own sustainability but also contribute to addressing global climate change. This finding diverges from Liu et al.’s [71] research, which focuses primarily on evaluating production operations via carbon trading prices. However, Conclusion 6 of this article centers on choosing the production mode according to the revenue per product unit. This is due to the cost-saving benefits of reducing energy consumption. It explains that low revenue per product unit enables high-carbon enterprises to maximize benefits through enhanced energy efficiency. In low-revenue scenarios, prioritizing cost-saving measures is crucial for maintaining profitability. Additionally, high-carbon enterprises are significant producers of carbon emissions and energy consumption, which adversely affect the environment. By enhancing energy efficiency, enterprises can minimize their carbon footprint, comply with environmental regulations, and improve their corporate image. When a production unit’s income increases, a high-carbon enterprise can maximize its profits by scaling down production. Meanwhile, Conclusion 6 is also validated by actual cases and data. For example, FYI Resource is a listed resource company based in Australia. It has adopted technologies that improve energy efficiency and achieve optimal results relative to other methods. It was found to produce alumina with 40% less energy and 50% of the GHG emissions of traditional methods [72]. In summary, enterprises with high carbon emissions adjust their energy usage and production scale based on income levels to maximize profits and income in different stages. This flexible policy helps maintain competitiveness and sustainable development in varying economic environments.

To enhance the policy impact of the findings, detailed and feasible implementation strategies can be developed based on the conclusions of this study. Strategy 1: Optimize production scale adjustment decisions. For high-carbon enterprises, when a reduction in production scale significantly reduces profits, companies should carefully assess the risks of drastic production cuts to avoid excessive reductions that could lead to a sharp decline in profits. Where necessary, consider reducing energy consumption through technological improvements or process optimization rather than drastically cutting production. For low-carbon enterprises, when reducing production scale, they should combine market demand and cost-benefit analysis to ensure that while maintaining sufficient output, they minimize carbon emissions as much as possible. Strategy 2: Increase investment in improving energy efficiency. For all enterprises, priority should be given to investing in technologies and equipment that can significantly improve energy efficiency, as this not only reduces carbon emissions but also leads to significant revenue growth. By improving energy efficiency, companies can increase production while maintaining or increasing profit levels. For low-carbon enterprises, when improving energy efficiency, they can take advantage of incentives from low-carbon policies to further reduce costs and increase revenue, thereby expanding production scale. Strategy 3: Balance renewable energy investment with cost control. For high-carbon enterprises, renewable energy use should be gradually increased within cost allowances, while closely monitoring its impact on production costs. Cost pressures can be mitigated through government subsidies, cooperative projects, or phased implementation. For low-carbon enterprises, the expansion of renewable energy can be considered a long-term goal, with a focus on improving energy efficiency in the short term, while preparing for future renewable energy expansion. Strategy 4: Prioritize the adoption of high-efficiency production models. For low-carbon enterprises, when the cost of changing production modes is low, companies should prioritize energy efficiency improvements as a way to achieve energy savings and emission reductions. This not only maintains low operating costs but also effectively increases market competitiveness. Strategy 5: Focus on improving energy efficiency. For low-carbon enterprises, when the cost of changing production modes is high and the revenue per unit product is low, companies should concentrate resources on improving the energy efficiency of existing production lines, avoiding unnecessary high costs due to excessive changes. This helps maintain economic benefits while sustaining low carbon emissions.

## 7. Conclusion

Energy efficiency and conservation based on carbon trading is a market-based mechanism for reducing greenhouse gas emissions. This mechanism aims to incentivize emissions reductions and energy savings, encouraging businesses and institutions to lower their carbon and other greenhouse gases. High-carbon enterprises can offset their carbon emissions by purchasing carbon emission rights from low-carbon enterprises, thereby achieving their carbon emission reduction goals. This trading mechanism promotes carbon emission reduction and the development of a low-carbon economy. This article proposes three methods for industrial enterprises to save energy and reduce emissions through carbon emission rights trading: reducing production scale, improving energy efficiency, or expanding renewable energy. To account for the changing decisions of low-carbon and high-carbon enterprises over time, a difference game model of the three methods is constructed. This study comprehensively synthesizes aspects of optimizing production scale, enhancing energy efficiency, and expanding the scope of renewable energy in carbon trading, thus filling a conspicuous void. Additionally, employing analytical methods such as game theory and optimization, this research thoroughly examines the large-scale implementation of innovative technologies. This enhances the contribution of innovative technologies to the carbon trading market and cultivates a more sustainable energy infrastructure. Finally, in the realm of operations management within carbon trading, this study ventures into a fundamentally undeveloped area by elucidating how to improve operational efficiency and management practices to achieve carbon trading objectives.

The study has also come up with very important and interesting findings. The results show that when the cost of changing the production mode and the income obtained from the production of unit product is large, the low-carbon enterprise can obtain the maximum benefit by reducing the production scale mode. Otherwise, low carbon enterprises can be maximized through improving energy efficiency mode. For both low-carbon and high-carbon enterprises, reducing production scale is the fastest way to enhance efficiency when the costs of energy conservation and emission reduction are substantial.

The research in this article can also be extended. For example, it is assumed that improving energy efficiency increases revenues and costs, and expanding the use of renewable energy can reduce costs. In future research, it can be assumed that improving energy efficiency does not increase cost, and expanding the use of renewable energy may increase cost, etc. for further research. At the same time, some gaps in the research can also be solved in future research. First, it is necessary to determine the specific standards adopted by industrial enterprises in energy conservation and emission reduction modes under different conditions. Second, in the context of carbon trading, the results of energy conservation and emission reduction should be transformed into practical policy recommendations for reference by low-carbon enterprises and high-carbon enterprises. Third, in the process of energy conservation and emission reduction in industrial enterprises, low-carbon enterprises and high-carbon enterprises should determine the order of action of relevant research, rather than taking action at the same time.

## Supporting information

S1 Appendix (DOCX)

S2 Appendix (DOCX)

S3 Appendix (DOCX)
