# Supplementary material for: Research on different modes of energy conservation and emission reduction: A differential game model based on carbon trading perspective
Source: PLoS One. 2024 Sep 4;19(9):e0309968. doi: 10.1371/journal.pone.0309968 (PMC11373867; doi:10.1371/journal.pone.0309968)
Supplement: S1 Appendix — (DOCX) [file pone.0309968.s001.docx]

**Appendix 1**

Take the derivatives of *F_R_*_1_ with respect to (13), and take the derivatives of *F_R_*_2_ with respect to (14), and set them equal to zero, we can get:

$F_{R1}^{\text{*}}\left(t\right)=\frac{b_{1}-b_{R}+\frac{\partial V_{R1}}{\partial x_{R1}}h_{1}}{c_{1}-c_{R}}$ (43)

$F_{R2}^{\text{*}}\left(t\right)=\frac{b_{2}-b_{R}+\frac{\partial V_{R2}}{\partial x_{R2}}h_{1}}{c_{2}-c_{R}}$ (44)

Substituting (43) into (13) and substituting (44) into (14), the article can get:

$\begin{matrix} \rho V_{R1}=\left(b_{1}-b_{R}\right)\frac{b_{1}-b_{R}+\frac{\partial V_{R1}}{\partial x_{R1}}h_{1}}{c_{1}-c_{R}}-\frac{c_{1}-c_{R}}{2}\left(\frac{b_{1}-b_{R}+\frac{\partial V_{R1}}{\partial x_{R1}}h_{1}}{c_{1}-c_{R}}\right)^{2}+\left(C_{a}-fb_{R}\right) \\ +lx_{R1}\left(t\right)+\frac{\partial V_{R1}}{\partial x_{R1}}\left[h_{1}\frac{b_{1}-b_{R}+\frac{\partial V_{R1}}{\partial x_{R1}}h_{1}}{c_{1}-c_{R}}-\delta x_{R1}\left(t\right)\right] \end{matrix}$ (45)

$\begin{matrix} \rho V_{R2}=\left(b_{2}-b_{R}\right)\frac{b_{2}-b_{R}+\frac{\partial V_{R2}}{\partial x_{R2}}h_{1}}{c_{2}-c_{R}}-\frac{c_{2}-c_{R}}{2}\left(\frac{b_{2}-b_{R}+\frac{\partial V_{R2}}{\partial x_{R2}}h_{1}}{c_{2}-c_{R}}\right)^{2}-\left(C_{a}-fb_{R}\right) \\ +lx_{R2}\left(t\right)+\frac{\partial V_{R2}}{\partial x_{R2}}\left[h_{2}\frac{b_{2}-b_{R}+\frac{\partial V_{R2}}{\partial x_{R2}}h_{1}}{c_{2}-c_{R}}-\delta x_{R2}\left(t\right)\right] \end{matrix}$ (46)

Let $V_{R1}^{*}=k_{1}x_{R1}+k_{2}$, $V_{R2}^{\text{*}}=k_{3}x_{R2}+k_{4}$, wherein, *k*_1_, *k*_2_, *k*_3_ and *k*_4_ are all constants. The parameters of the optimal social welfare function can be obtained by calculation as follows:

$\left\{\begin{matrix} k_{1}=\frac{l}{\rho+\delta} \\ k_{2}=\frac{1}{\rho}\left(b_{1}-b_{R}\right)\frac{b_{1}-b_{R}+\frac{l}{\rho+\delta}h_{1}}{c_{1}-c_{R}}-\frac{c_{1}-c_{R}}{2}\frac{1}{\rho}\left(\frac{b_{1}-b_{R}+\frac{l}{\rho+\delta}h_{1}}{c_{1}-c_{R}}\right)^{2}+\frac{1}{\rho}\left(C_{a}-fb_{R}\right) \\ +\frac{1}{\rho}\frac{l}{\rho+\delta}h_{1}\frac{b_{1}-b_{R}+\frac{l}{\rho+\delta}h_{1}}{c_{1}-c_{R}} \end{matrix}\right.$ (47)

$\left\{\begin{matrix} k_{3}=\frac{l}{\rho+\delta} \\ k_{4}=\frac{1}{\rho}\left(b_{2}-b_{R}\right)\frac{b_{2}-b_{R}+\frac{l}{\rho+\delta}h_{1}}{c_{2}-c_{R}}-\frac{1}{\rho}\frac{c_{2}-c_{R}}{2}\left(\frac{b_{2}-b_{R}+\frac{l}{\rho+\delta}h_{1}}{c_{2}-c_{R}}\right)^{2}-\frac{1}{\rho}\left(C_{a}-fb_{R}\right) \\ +\frac{1}{\rho}\frac{l}{\rho+\delta}h_{2}\frac{b_{2}-b_{R}+\frac{l}{\rho+\delta}h_{1}}{c_{2}-c_{R}} \end{matrix}\right.$ (48)

Therefore, it can be concluded that:

$\begin{matrix} V_{R1}^{*}=\frac{l}{\rho+\delta}x_{R1}+\frac{1}{\rho}\left(b_{1}-b_{R}\right)\frac{b_{1}-b_{R}+\frac{l}{\rho+\delta}h_{1}}{c_{1}-c_{R}}-\frac{c_{1}-c_{R}}{2}\frac{1}{\rho}\left(\frac{b_{1}-b_{R}+\frac{l}{\rho+\delta}h_{1}}{c_{1}-c_{R}}\right)^{2}+\frac{1}{\rho}\left(C_{a}-fb_{R}\right) \\ +\frac{1}{\rho}\frac{l}{\rho+\delta}h_{1}\frac{b_{1}-b_{R}+\frac{l}{\rho+\delta}h_{1}}{c_{1}-c_{R}} \end{matrix}$ (49)

$\begin{matrix} V_{R2}^{\text{*}}=\frac{l}{\rho+\delta}x_{R2}+\frac{1}{\rho}\left(b_{2}-b_{R}\right)\frac{b_{2}-b_{R}+\frac{l}{\rho+\delta}h_{1}}{c_{2}-c_{R}}-\frac{1}{\rho}\frac{c_{2}-c_{R}}{2}\left(\frac{b_{2}-b_{R}+\frac{l}{\rho+\delta}h_{1}}{c_{2}-c_{R}}\right)^{2}-\frac{1}{\rho}\left(C_{a}-fb_{R}\right) \\ +\frac{1}{\rho}\frac{l}{\rho+\delta}h_{2}\frac{b_{2}-b_{R}+\frac{l}{\rho+\delta}h_{1}}{c_{2}-c_{R}} \end{matrix}$ (50)

In this case,

$F_{R1}^{\text{*}}\left(t\right)=\frac{b_{1}-b_{R}+\frac{l}{\rho+\delta}h_{1}}{c_{1}-c_{R}}$ (51)

$F_{R2}^{\text{*}}\left(t\right)=\frac{b_{2}-b_{R}+\frac{l}{\rho+\delta}h_{1}}{c_{2}-c_{R}}$ (52)
