# Supplementary material for: Research on different modes of energy conservation and emission reduction: A differential game model based on carbon trading perspective
Source: PLoS One. 2024 Sep 4;19(9):e0309968. doi: 10.1371/journal.pone.0309968 (PMC11373867; doi:10.1371/journal.pone.0309968)
Supplement: S2 Appendix — (DOCX) [file pone.0309968.s002.docx]

**Appendix 2**

Take the derivatives of *F_I_*_1_ with respect to (15), and take the derivatives of *F_I_*_2_ with respect to (16), and set them equal to zero, the articlee can get:

$F_{I1}^{\text{*}}\left(t\right)=\frac{b_{1}+b_{I}+\frac{\partial V_{I1}}{\partial x_{I1}}h_{1}}{c_{1}+c_{I}}$ (53)

$F_{I2}^{\text{*}}\left(t\right)=\frac{b_{2}+b_{I}+\frac{\partial V_{I2}}{\partial x_{I2}}h_{1}}{c_{2}+c_{I}}$ (54)

Substituting (53) into (15) and substituting (54) into (16), we can get:

$\begin{matrix} \rho V_{I1}=\left(b_{1}+b_{I}\right)\frac{b_{1}+b_{I}+\frac{\partial V_{I1}}{\partial x_{I1}}h_{1}}{c_{1}+c_{I}}-\frac{c_{1}+c_{I}}{2}\left(\frac{b_{1}+b_{I}+\frac{\partial V_{I1}}{\partial x_{I1}}h_{1}}{c_{1}+c_{I}}\right)^{2}+C_{a}+C_{I} \\ +lx_{I1}\left(t\right)+\frac{\partial V_{I1}}{\partial x_{I1}}\left[h_{1}\frac{b_{1}+b_{I}+\frac{\partial V_{I1}}{\partial x_{I1}}h_{1}}{c_{1}+c_{I}}-\delta x_{I1}\left(t\right)\right] \end{matrix}$ (55)

$\begin{matrix} \rho V_{I2}=\left(b_{2}\text{+}b_{I}\right)\frac{b_{2}+b_{I}+\frac{\partial V_{I2}}{\partial x_{I2}}h_{1}}{c_{2}+c_{I}}-\frac{c_{2}+c_{I}}{2}\left(\frac{b_{2}+b_{I}+\frac{\partial V_{I2}}{\partial x_{I2}}h_{1}}{c_{2}+c_{I}}\right)^{2}-C_{a}+C_{I} \\ +lx_{I2}\left(t\right)+\frac{\partial V_{I2}}{\partial x_{I2}}\left[h_{2}\frac{b_{2}+b_{I}+\frac{\partial V_{I2}}{\partial x_{I2}}h_{1}}{c_{2}+c_{I}}-\delta x_{I2}\left(t\right)\right] \end{matrix}$ (56)

Let $V_{I1}^{*}=k_{5}x_{I1}+k_{6}$,$V_{I2}^{\text{*}}=k_{7}x_{I2}+k_{8}$, wherein, *k*_5_, *k*_6_, *k*_7_ and *k*_8_ are all constants. The parameters of the optimal social welfare function can be obtained by calculation as follows:

$\left\{\begin{matrix} k_{5}=\frac{l}{\rho+\delta} \\ k_{6}=\frac{1}{\rho}\left(b_{1}+b_{I}\right)\frac{b_{1}+b_{I}+\frac{l}{\rho+\delta}h_{1}}{c_{1}+c_{I}}-\frac{c_{1}+c_{I}}{2}\frac{1}{\rho}\left(\frac{b_{1}+b_{I}+\frac{l}{\rho+\delta}h_{1}}{c_{1}+c_{I}}\right)^{2}+\frac{1}{\rho}C_{a}+\frac{1}{\rho}C_{I} \\ +\frac{1}{\rho}\frac{l}{\rho+\delta}h_{1}\frac{b_{1}+b_{I}+\frac{l}{\rho+\delta}h_{1}}{c_{1}+c_{I}} \end{matrix}\right.$ (57)

$\left\{\begin{matrix} k_{7}=\frac{l}{\rho+\delta} \\ k_{8}=\frac{1}{\rho}\left(b_{2}\text{+}b_{I}\right)\frac{b_{2}+b_{I}+\frac{l}{\rho+\delta}h_{1}}{c_{2}+c_{I}}-\frac{c_{2}+c_{I}}{2}\frac{1}{\rho}\left(\frac{b_{2}+b_{I}+\frac{l}{\rho+\delta}h_{1}}{c_{2}+c_{I}}\right)^{2}-\frac{1}{\rho}C_{a}+\frac{1}{\rho}C_{I} \\ +\frac{l}{\rho+\delta}\frac{1}{\rho}h_{2}\frac{b_{2}+b_{I}+\frac{l}{\rho+\delta}h_{1}}{c_{2}+c_{I}} \end{matrix}\right.$ (58)

Therefore, it can be concluded that:

$\begin{matrix} V_{I1}^{*}=\frac{l}{\rho+\delta}x_{I1}+\frac{1}{\rho}\left(b_{1}+b_{I}\right)\frac{b_{1}+b_{I}+\frac{l}{\rho+\delta}h_{1}}{c_{1}+c_{I}}-\frac{c_{1}+c_{I}}{2}\frac{1}{\rho}\left(\frac{b_{1}+b_{I}+\frac{l}{\rho+\delta}h_{1}}{c_{1}+c_{I}}\right)^{2}+\frac{1}{\rho}C_{a}+\frac{1}{\rho}C_{I} \\ +\frac{1}{\rho}\frac{l}{\rho+\delta}h_{1}\frac{b_{1}+b_{I}+\frac{l}{\rho+\delta}h_{1}}{c_{1}+c_{I}} \end{matrix}$ (59)

$\begin{matrix} V_{I2}^{\text{*}}=\frac{l}{\rho+\delta}x_{I2}+\frac{1}{\rho}\left(b_{2}\text{+}b_{I}\right)\frac{b_{2}+b_{I}+\frac{l}{\rho+\delta}h_{1}}{c_{2}+c_{I}}-\frac{c_{2}+c_{I}}{2}\frac{1}{\rho}\left(\frac{b_{2}+b_{I}+\frac{l}{\rho+\delta}h_{1}}{c_{2}+c_{I}}\right)^{2}-\frac{1}{\rho}C_{a}+\frac{1}{\rho}C_{I} \\ +\frac{l}{\rho+\delta}\frac{1}{\rho}h_{2}\frac{b_{2}+b_{I}+\frac{l}{\rho+\delta}h_{1}}{c_{2}+c_{I}} \end{matrix}$ (60)

In this case,

$F_{I1}^{\text{*}}\left(t\right)=\frac{b_{1}+b_{I}+\frac{l}{\rho+\delta}h_{1}}{c_{1}+c_{I}}$ (61)

$F_{I2}^{\text{*}}\left(t\right)=\frac{b_{2}+b_{I}+\frac{l}{\rho+\delta}h_{1}}{c_{2}+c_{I}}$ (62)
