# Supplementary material for: Research on different modes of energy conservation and emission reduction: A differential game model based on carbon trading perspective
Source: PLoS One. 2024 Sep 4;19(9):e0309968. doi: 10.1371/journal.pone.0309968 (PMC11373867; doi:10.1371/journal.pone.0309968)
Supplement: S3 Appendix — (DOCX) [file pone.0309968.s003.docx]

**Appendix 3**

Take the derivatives of *F_E_*_1_ with respect to (17), and take the derivatives of *F_E_*_2_ with respect to (18), and set them equal to zero, the article can get:

$F_{E1}^{\text{*}}\left(t\right)=\frac{b_{1}+\frac{\partial V_{E1}}{\partial x_{E1}}h_{1}}{c_{1}+c_{E}}$ (63)

$F_{E2}^{\text{*}}\left(t\right)=\frac{b_{2}+\frac{\partial V_{E2}}{\partial x_{E2}}h_{2}}{c_{2}+c_{E}}$ (64)

Substituting (63) into (17) and substituting (64) into (18), we can get:

$\begin{matrix} \rho V_{E1}=b_{1}\frac{b_{1}+\frac{\partial V_{E1}}{\partial x_{E1}}h_{1}}{c_{1}+c_{E}}-\frac{c_{1}+c_{E}}{2}\left(\frac{b_{1}+\frac{\partial V_{E1}}{\partial x_{E1}}h_{1}}{c_{1}+c_{E}}\right)^{2}+C_{a}+C_{E}+lx_{E1}\left(t\right) \\ +\frac{\partial V_{E1}}{\partial x_{E1}}\left[h_{1}\frac{b_{1}+\frac{\partial V_{E1}}{\partial x_{E1}}h_{1}}{c_{1}+c_{E}}-\delta x_{E1}\left(t\right)\right] \end{matrix}$ (65)

$\begin{matrix} \rho V_{E2}=b_{2}\frac{b_{2}+\frac{\partial V_{E2}}{\partial x_{E2}}h_{2}}{c_{2}+c_{E}}-\frac{c_{2}+c_{E}}{2}\left(\frac{b_{2}+\frac{\partial V_{E2}}{\partial x_{E2}}h_{2}}{c_{2}+c_{E}}\right)^{2}-C_{a}+C_{E}+lx_{E2}\left(t\right) \\ +\frac{\partial V_{E2}}{\partial x_{E2}}\left[h_{2}\frac{b_{2}+\frac{\partial V_{E2}}{\partial x_{E2}}h_{2}}{c_{2}+c_{E}}-\delta x_{E2}\left(t\right)\right] \end{matrix}$ (66)

Let $V_{E1}^{*}=k_{9}x_{E1}+k_{10}$,$V_{E2}^{\text{*}}=k_{11}x_{E2}+k_{12}$, wherein, *k*_9_, *k*_10_, *k*_11_ and *k*_12_ are all constants. The parameters of the optimal social welfare function can be obtained by calculation as follows:

$\left\{\begin{matrix} k_{9}=\frac{l}{\rho+\delta} \\ k_{10}=\frac{1}{\rho}b_{1}\frac{b_{1}+\frac{l}{\rho+\delta}h_{1}}{c_{1}+c_{E}}-\frac{c_{1}+c_{E}}{2}\frac{1}{\rho}\left(\frac{b_{1}+\frac{l}{\rho+\delta}h_{1}}{c_{1}+c_{E}}\right)^{2}+\frac{1}{\rho}C_{a}+\frac{1}{\rho}C_{E}+\frac{l}{\rho+\delta}\frac{1}{\rho}h_{1}\frac{b_{1}+\frac{l}{\rho+\delta}h_{1}}{c_{1}+c_{E}} \end{matrix}\right.$ (67)

$\left\{\begin{matrix} k_{11}=\frac{l}{\rho+\delta} \\ k_{12}=\frac{1}{\rho}b_{2}\frac{b_{2}+\frac{l}{\rho+\delta}h_{2}}{c_{2}+c_{E}}-\frac{c_{2}+c_{E}}{2}\frac{1}{\rho}\left(\frac{b_{2}+\frac{l}{\rho+\delta}h_{2}}{c_{2}+c_{E}}\right)^{2}-\frac{1}{\rho}C_{a}+\frac{1}{\rho}C_{E}+\frac{l}{\rho+\delta}\frac{1}{\rho}h_{2}\frac{b_{2}+\frac{l}{\rho+\delta}h_{2}}{c_{2}+c_{E}} \end{matrix}\right.$(68)

Therefore, it can be concluded that:

$\begin{matrix} V_{E1}^{*}=\frac{l}{\rho+\delta}x_{E1}+\frac{1}{\rho}b_{1}\frac{b_{1}+\frac{l}{\rho+\delta}h_{1}}{c_{1}+c_{E}}-\frac{c_{1}+c_{E}}{2}\frac{1}{\rho}\left(\frac{b_{1}+\frac{l}{\rho+\delta}h_{1}}{c_{1}+c_{E}}\right)^{2}+\frac{1}{\rho}C_{a}+\frac{1}{\rho}C_{E} \\ +\frac{l}{\rho+\delta}\frac{1}{\rho}h_{1}\frac{b_{1}+\frac{l}{\rho+\delta}h_{1}}{c_{1}+c_{E}} \end{matrix}$ (69)

$\begin{matrix} V_{E2}^{\text{*}}=\frac{l}{\rho+\delta}x_{E2}+\frac{1}{\rho}b_{2}\frac{b_{2}+\frac{l}{\rho+\delta}h_{2}}{c_{2}+c_{E}}-\frac{c_{2}+c_{E}}{2}\frac{1}{\rho}\left(\frac{b_{2}+\frac{l}{\rho+\delta}h_{2}}{c_{2}+c_{E}}\right)^{2}-\frac{1}{\rho}C_{a}+\frac{1}{\rho}C_{E} \\ +\frac{l}{\rho+\delta}\frac{1}{\rho}h_{2}\frac{b_{2}+\frac{l}{\rho+\delta}h_{2}}{c_{2}+c_{E}} \end{matrix}$ (70)

In this case,

$F_{E1}^{\text{*}}\left(t\right)=\frac{b_{1}+\frac{l}{\rho+\delta}h_{1}}{c_{1}+c_{E}}$ (71)

$F_{E2}^{\text{*}}\left(t\right)=\frac{b_{2}+\frac{l}{\rho+\delta}h_{2}}{c_{2}+c_{E}}$ (72)
